# Allelic variations of *WAK106‐E2Fa‐DPb1‐UGT74E2* module regulate fibre properties in *Populus tomentosa*

**DOI:** 10.1111/pbi.14239

**Published:** 2023-11-21

**Authors:** Dan Wang, Mingyang Quan, Shitong Qin, Yuanyuan Fang, Liang Xiao, Weina Qi, Yongsen Jiang, Jiaxuan Zhou, Mingyue Gu, Yicen Guan, Qingzhang Du, Qing Liu, Yousry A. El‐Kassaby, Deqiang Zhang

**Affiliations:** ^1^ State Key Laboratory of Tree Genetics and Breeding, College of Biological Sciences and Technology Beijing Forestry University Beijing China; ^2^ National Engineering Laboratory for Tree Breeding, College of Biological Sciences and Technology Beijing Forestry University Beijing China; ^3^ Key Laboratory of Genetics and Breeding in Forest Trees and Ornamental Plants, Ministry of Education, College of Biological Sciences and Technology Beijing Forestry University Beijing China; ^4^ CSIRO Agriculture and Food Black Mountain Canberra ACT Australia; ^5^ Department of Forest and Conservation Sciences, Faculty of Forestry, Forest Sciences Centre University of British Columbia Vancouver BC Canada

**Keywords:** co‐expression, association study, *PtoDPb1*, allelic variations, cellulose, *Populus*

## Abstract

Wood formation, intricately linked to the carbohydrate metabolism pathway, underpins the capacity of trees to produce renewable resources and offer vital ecosystem services. Despite their importance, the genetic regulatory mechanisms governing wood fibre properties in woody plants remain enigmatic. In this study, we identified a pivotal module comprising 158 high‐priority core genes implicated in wood formation, drawing upon tissue‐specific gene expression profiles from 22 *Populus* samples. Initially, we conducted a module‐based association study in a natural population of 435 *Populus tomentosa*, pinpointing *PtoDPb1* as the key gene contributing to wood formation through the carbohydrate metabolic pathway. Overexpressing *PtoDPb1* led to a 52.91% surge in cellulose content, a reduction of 14.34% in fibre length, and an increment of 38.21% in fibre width in transgenic poplar. Moreover, by integrating co‐expression patterns, RNA‐sequencing analysis, and expression quantitative trait nucleotide (eQTN) mapping, we identified a *PtoDPb1*‐mediated genetic module of *PtoWAK106*‐*PtoDPb1*‐*PtoE2Fa‐PtoUGT74E2* responsible for fibre properties in *Populus*. Additionally, we discovered the two *PtoDPb1* haplotypes that influenced protein interaction efficiency between PtoE2Fa‐PtoDPb1 and PtoDPb1‐PtoWAK106, respectively. The transcriptional activation activity of the PtoE2Fa‐PtoDPb1 haplotype‐1 complex on the promoter of *PtoUGT74E2* surpassed that of the PtoE2Fa‐PtoDPb1 haplotype‐2 complex. Taken together, our findings provide novel insights into the regulatory mechanisms of fibre properties in *Populus*, orchestrated by *PtoDPb1*, and offer a practical module for expediting genetic breeding in woody plants via molecular design.

## Introduction

The secondary growth of perennial woody plants stimulates a progressive increase in stem growth and wood formation, which is essential for the production of cellulose, hemicelluloses, and lignin (Déjardin *et al*., 2010; Du and Groover, 2010). As the most abundant biological polymer, cellulose serves as a vital raw material in various industries, including textiles, pulp, and paper (Porth and El‐Kassaby, 2015; Ragauskas *et al*., 2014; Studer *et al*., 2011). Cellulose's linear structure, comprised of β‐1,4‐linked D‐glucopyranose molecules, plays a pivotal role in the synthesis and remodelling of carbohydrates within the secondary cell wall of woody plants, leading to highly efficient cellulose biosynthesis (Read and Bacic, 2002). Uridine diphosphate (UDP)–glucose and fructose are catalysed by the UDP reaction, which directly augments the efficiency of cellulose biosynthesis, paving the way for improved lignocellulose utilization (Babb and Haigler, 2001). For instance, the overexpression of the gene encoding UDP‐glucose pyrophosphorylase (UGPase) in *Larix gmelinii* was shown to boost plant vegetative growth by increasing soluble sugars and cellulose contents, as well as thickening parenchymal cell walls (Li *et al*., 2014). Therefore, UDP‐sugar is instrumental in cellulose synthesis in woody plants. Its catalytic impact can directly amplify the efficiency of cellulose biosynthesis, thereby fostering the development and production of lignocellulosic biomass.

Previous studies have underscored that complex transcriptional regulation is fundamental to the various developmental stages of wood formation (Chen *et al*., 2019; Hori *et al*., 2020; Qin *et al*., 2020). Two primary transcription factor (TF) families, *that is*, NAC and MYB, play key roles in the hierarchical regulatory networks underlying secondary cell wall (SCW) formation (Nakano *et al*., 2015). In this network, NST1‐3 were identified to regulate a battery of downstream transcription factors, which in turn activate the biosynthetic genes for secondary wall deposition (Kubo *et al*., 2005; Mitsuda *et al*., 2007; Zhong *et al*., 2008). In addition, numerous TFs can impact SCW biosynthesis by directly modulating genes that control carbohydrate biosynthesis. For instance, the expression of genes responsible for the cellulose and hemicellulose biosynthesis, such as *CESA7*, *CESA8*, *IRX8*, and *IRX9*, was induced by VND7 and MYB46 (Kim *et al*., 2012, 2013; Kumar *et al*., 2016; Yamaguchi *et al*., 2011). Notably, the E2‐promoter binding factor and DRTF‐1‐polypeptide (E2F‐DP) complex has been identified as a top‐tier regulator in the hierarchical genetic networks of SCW in *Arabidopsis*, governing carbohydrate genes like *CESA4*, *CESA7*, *IRX7*, and *GUX2* (Taylor‐Teeples *et al*., 2015). However, the intricate transcriptional mechanism by which the E2F‐DP complex regulates SCW in perennial woody plants remains largely shrouded in mystery. This gap in our understanding necessitates further research to fully unravel the potential of these genetic regulatory networks for enhancing wood fibre properties.

The traditionally recognized role of the E2F‐DP complex pertains to its pivotal influence on the cell cycle transition, as highlighted by Sozzani *et al*. (2006). Intriguingly, beyond its canonical function, the E2F‐DP complex demonstrates its involvement in a myriad of biological roles, such as orchestrating the intricate relationship between cell proliferation, differentiation, and growth (Magyar *et al*., 2005; Stevens and La Thangue, 2003). In *Arabidopsis thaliana* genome, a trio of E2F members (*E2Fa*, *E2Fb*, and *E2Fc*) and a duo of DP members (*DPa* and *DPb*) have been unearthed (Vandepoele *et al*., 2005). Notably, both E2Fa‐DP and E2Fb‐DP complexes can potentially function as transcriptional activators, driving the expression of reporter genes that contain E2F/DP consensus *cis*‐acting elements (Mariconti *et al*., 2002; Stevens *et al*., 2002). It was reported that transient overexpression of *E2Fa* and *DPa* can induce ectopic cell division within plant cells (De Veylder *et al*., 2002; Kosugi and Ohashi, 2003; Rossignol *et al*., 2002). Furthermore, ectopic co‐expressions of *E2Fc* and *DPb* can result in severe developmental aberrations in *Arabidopsis* (Del Pozo *et al*., 2006). Considering the indispensable role of DP as an essential partner of the E2F‐DP complex (Magyar *et al*., 2000; Mariconti *et al*., 2002), surprisingly few studies have delved into the mechanism through which natural variations of DP impact their interactive dynamics. Therefore, an extensive exploration of the interplay between E2F and DP is of vital importance, which will enrich our understanding of the comprehensive regulatory network governing wood formation in woody plants.

Association studies have emerged as instrumental tools in dissecting gene modules that regulate traits of interest. These studies have been successful in unearthing genetic variants that engender phenotypic divergences in an eclectic array of tree species, including *Populus tomentosa*, *Picea abies*, and *Pinus taeda* (Caré *et al*., 2020; Lu *et al*., 2017; Xiao *et al*., 2019). Of vital significance is the comprehension of the genetic underpinnings of natural variations in complex traits at the transcriptional level, which serves as a vital cog in the machinery of genetic information for these traits. This understanding aptly complements the information gleaned from association studies (Quan *et al*., 2018; Xiao *et al*., 2019). eQTN mapping offers a potent tool to decode single nucleotide polymorphisms (SNPs) that influence gene expression, thereby forging mechanistic bridges between genotype and phenotype (Song *et al*., 2021; Zhao *et al*., 2021). Crucially, co‐expression networks embody gene clusters that display strikingly congruent expression profiles, making them susceptible to shared biological regulatory pathways (Eisen *et al*., 1998). These networks offer panoramic insights into the genetic architecture of quantitative traits in *Populus* (Yang *et al*., 2011). Thus, a strategic blend of association studies and transcriptional analysis holds promise to effectively pinpoint the hierarchical regulatory network that undergirds SCW biosynthesis in plants.

Here, we employed co‐expression analysis combined with a module‐based association study to identify a high‐priority gene, *PtoDPb1*, which plays a significant role in wood formation within the natural population of *P. tomentosa*. We further discerned two haplotype groups stemming from *PtoDPb1*, comprising three non‐synonymous variants that were linked with the holocellulose content (HC) and 6‐bp insertion and/or deletion (InDels) (minor allele frequency (MAF) > 0.05), again within the natural population of *P. tomentosa*. Utilizing eQTN mapping and expression analysis, we were able to identify the upstream regulator *PtoWAK106* as well as the downstream gene *PtoUGT74E2*, both of which are associated with *PtoDPb1*. Interestingly, individuals harbouring the high‐HC haplotype of *PtoDPb1* exhibited enhanced protein interaction efficiency between PtoE2Fa‐PtoDPb1 and PtoWAK106‐PtoDPb1, respectively. Moreover, these individuals also displayed improved transcriptional activation activity of the PtoE2Fa‐PtoDPb1 complex targeting the *PtoUGT74E2* promoter. Collectively, our findings reveal a novel regulatory module, *PtoWAK106*‐*PtoDPb1*‐*PtoE2Fa*‐*PtoUGT74E2*, that orchestrates the regulation of fibre properties in *Populus*.

## Results

### Identification of key gene *PtoDPb1* affecting wood properties and carbohydrate metabolism in *Populus*

**Figure 1:**
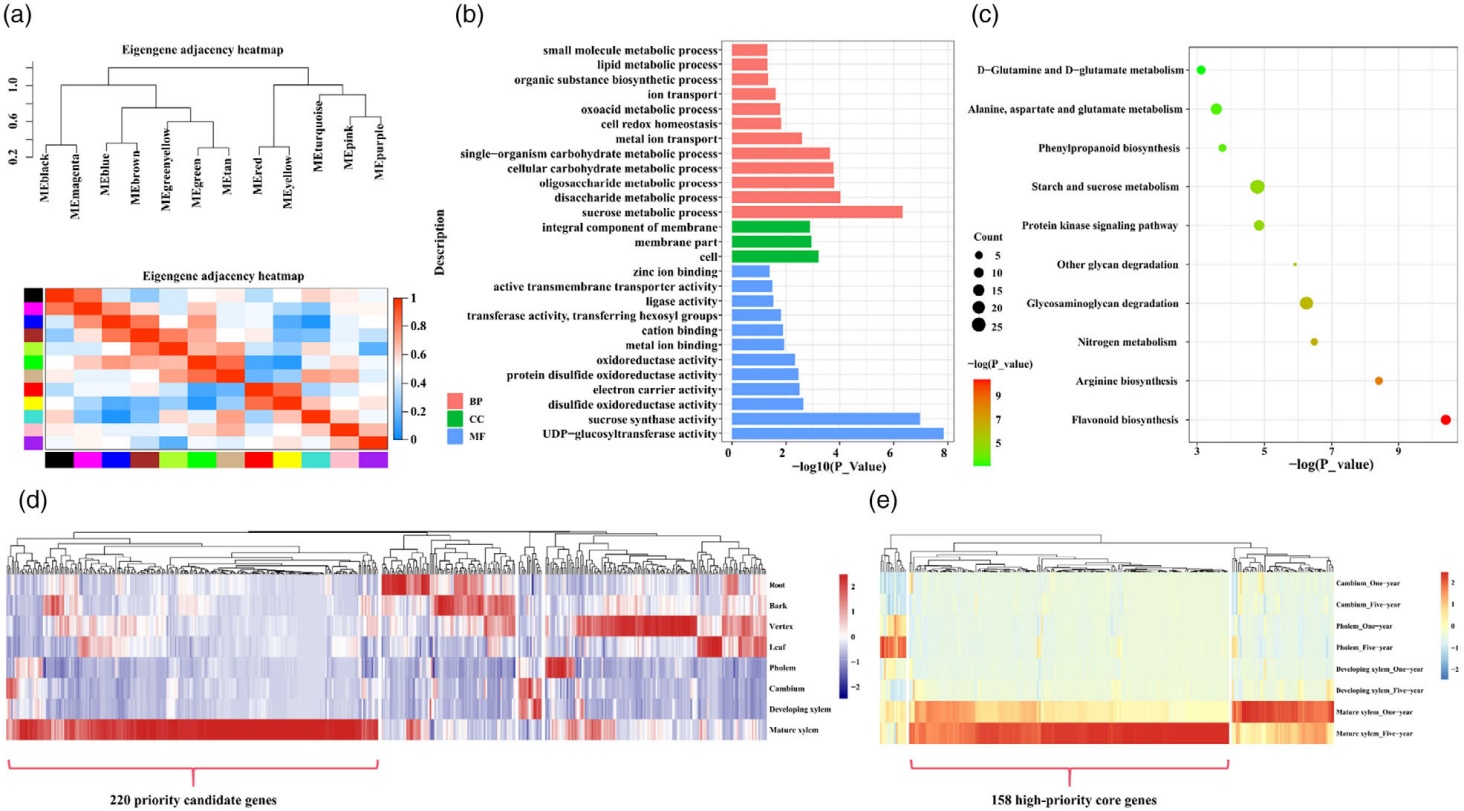
Identification and annotation of the green module associated with wood formation via weighted gene co‐expression network analysis (WGCNA). (a) A dendrogram, representing a hierarchical cluster tree, unveils twelve modules of co‐expressed genes. Each module is represented by a major branch in this gene tree. The lower panel shows these modules in their respective colour assignments. The curve altitude signifies the correlation coefficient of modules, while the colour variations designate different modules. (b) Gene Ontology (GO) enrichment for the green module. The abbreviations BP, CC, and MF correspond to biological process, cellular component, and molecular function, respectively. (c) Kyoto Encyclopedia of Genes and Genomes (KEGG) analysis for the green module. The sphere's size is proportional to gene count, whereas the hue of the sphere is indicative of the *P* value's magnitude. (d) The expression patterns of 453 high‐connectivity genes in the green module across various tissues in *Populus tomentosa*. (e) The expression patterns of 220 priority candidate genes from the green module within vascular tissues of one and 5‐year‐old *P. tomentosa*.

In our pursuit of a holistic understanding of the gene expression networks during wood formation in *Populus*, we identified 12 co‐expressed modules (Figure 1a) through the application of weighted gene co‐expression network analysis (WGCNA) on 22 sets of transcriptome data derived from the vascular tissue of *P. tomentosa* and *P. trichocarpa* (Table S1). To pinpoint the core modules implicated in wood formation, we deployed functional enrichment analysis by Gene Ontology (GO). This analysis revealed that the green module was significantly enriched in glucosyltransferase activity and carbohydrate metabolism processes (Dataset S1 and Figure 1b). Furthermore, Kyoto Encyclopaedia of Genes and Genomes (KEGG) analysis indicated that the green module was enriched in pathways related to flavonoid biosynthesis and phenylpropane metabolism (Dataset S2 and Figure 1c). Thus, the green module emerged as a likely key player in the orchestration of the wood formation. From the green module, we then examined the expression patterns of 453 genes exhibiting high connectivity (connectivity >10, weight >0.2, and gene Module Membership >0.8) (Dataset S3). This excise revealed that 220 priority candidate genes exhibited specific expression in the mature xylem of a 1‐year‐old tree (Figure 1d and Dataset S4). Furthermore, 158 of these genes were also robustly expressed in the mature xylem of 5‐year‐old trees (Figure 1e and Dataset S5), thereby establishing them as high‐priority core genes for subsequent analyses.

**Figure 2:**
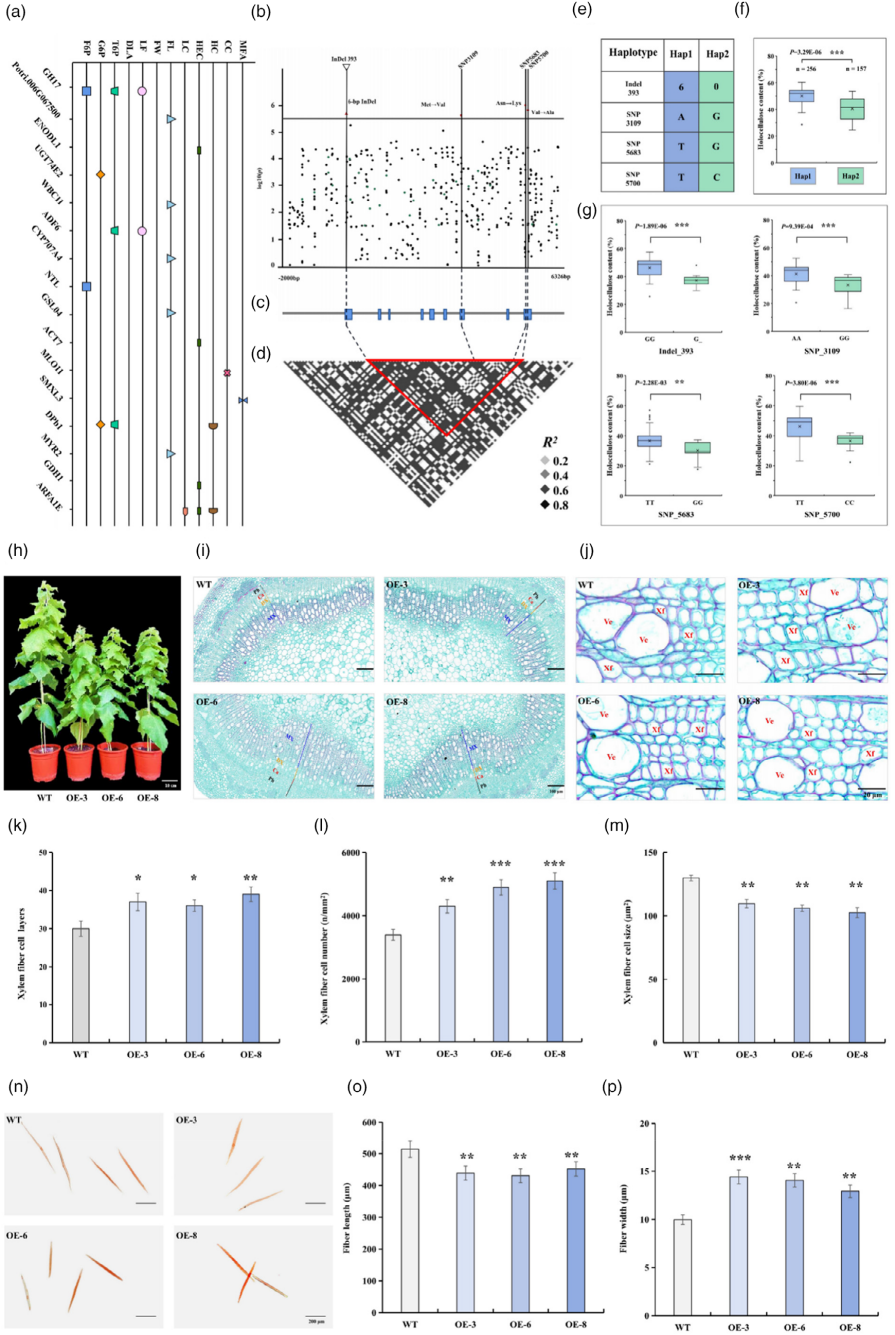
The genetic effects of natural variations in *PtoDPb1* and the phenotypes in *PtoDPb1*‐overexpressing lines. (a) The green module‐based association study (*P* ≤ 5.81E−05). Each point corresponds to a trait, with multiple points indicating multiple single nucleotide polymorphisms (SNPs) associated with a single gene. The x‐axis represents traits, while the y‐axis denotes the genes. CC, α‐cellulose content; DLA, DL‐Arabinose; F6P, D‐Fructose 6‐phosphate; FL, fibre length; FW, fibre width; G6P, Glucose 6‐phosphate; HC, holocellulose content; HEC, hemicellulose content; LC, lignin content; LF, L‐Fucose; MFA, microfibril angle; T6P, Trehalose 6‐phosphate. (b) The association analysis of genetic variations in *PtoDPb1* vis‐a‐vis holocellulose content (HC). Black dots denote SNPs, while green dots represent insertions and/or deletions (InDels). Three significant nonsynonymous variants and 6‐bp InDels are highlighted by red dots and triangle, respectively. (c) The gene structure of PtoDPb1, with blue rectangles indicating transcribed sequences and grey lines signifying non‐transcribed sequences. (d) The pattern of pairwise linkage disequilibrium (LD) of three nonsynonymous variants and 6‐bp InDels in *PtoDPb1*. Dotted lines, respectively, connect these variants and InDels points to their paired LD plots. The pronounced LD of these changes is underscored by a red line. (e) Two distinct haplotypes of *PtoDPb1*, identified among natural variations, were categorized in *Populus tomentosa*. (f, g) The HC distribution for each haplotype group (f) and each individual SNP site (g) are depicted via a box plot. In these box plots, median values are represented by centre marks, variability beyond the upper and lower quartiles is shown by solid lines, and outliers are marked by dots. ‘*n*’ denotes the count of genotypes within each haplotype group. (h) Phenotypic representations of wild type (WT), *35s: PtoDPb1*‐3 (OE‐3), *35s:PtoDPb1*‐6 (OE‐6), and *35s:PtoDPb1*‐8 (OE‐8) plants. (i, j) Histochemical staining in the stem sections of WT, OE‐3, OE‐6, and OE‐8 plants. Labels MX, DX, Ph, Ca, Ve, and Xf refer to mature xylem, developing xylem, phloem, cambium, vessel and xylem fibre cell, respectively. (k–m) Quantitative measurement of phloem fibre cell layers (k), number (l), and size (m) of WT, OE‐3, OE‐6, and OE‐8 plants. (n–p) Fibre length and fibre width measurement of WT, OE‐3, OE‐6, and OE‐8 plants. Error bars depict standard deviations (SD). Significant differences were determined using *t*‐test, *P < 0.05, **P < 0.01, ***P < 0.001.

To unravel the metabolic pathways engaged by the 158 high‐priority core genes in wood formation in *P. tomentosa*, we conducted a module‐based association study. This study utilized 17 223 SNPs derived from 158 genes (MAF > 0.05), which were identified from full‐length genomic sequences based on 435 re‐sequencing individuals from the natural population of *P. tomentosa*. We examined the associations of these SNPs with seven wood property traits and 15 metabolic traits, which included five metabolites for each of carbohydrate, lignin, and flavonoid. From our analysis, 35 significant SNPs were found to exhibit substantial associations with wood property and carbohydrate metabolic traits at *P* ≤ 5.81E−05 (employing a Bonferroni correction, *P* = 1/*n*; Table S2). Interestingly, we found no SNPs that were significantly associated with either lignin and/or flavonoid metabolites at *P* ≤ 5.81E−05. These significant associations corresponded to 25 unique SNPs across 10 traits, including 21 and nine significant associations for wood properties and carbohydrate traits, respectively (Table S2). These significantly associated SNPs could be traced back to 16 genes involved in various functions, with some directly impacting the wood formation process (Figure 2a and Table S2). Among these 16 genes, only *Potri.016G093200*, encoding a DP protein (*PtoDPb1*), exhibited associations with both wood quality and carbohydrate metabolite traits. Five significantly associated SNPs in *PtoDPb1*, namely, Chr16_6268473, Chr16_6269705, Chr16_6273232, Chr16_6275806, and Chr16_6275823, demonstrated pleiotropy for three traits: HC, trehalose 6‐phosphate (T6P), and glucose 6‐phosphate (G6P). These results were aligned with the elevated expression level of *PtoDPb1* observed in the mature xylem of poplar (Figure S1). Therefore, it is plausible to consider *PtoDPb1* as the key gene warranting further functional investigation.

To elucidate the genetic effects of *PtoDPb1* alleles, we re‐sequenced an 8.3‐kb genomic DNA fragment, which encompasses its entire coding region and 2‐kb upstream promoter, in 435 *P. tomentosa* accessions. This revealed a total of 389 SNPs and 28 InDels (Figure 2b). Among these, we identified five variants within the coding region, comprising a 6‐bp InDels and four non‐synonymous variants (MAF ≥ 0.05) of *PtoDPb1* in *P. tomentosa* (Figure S2). A candidate gene‐based association study detected three significantly associated non‐synonymous variants in the heterodimerization domain (HD) region of *PtoDPb1*: Chr16_6273232 (*P* = 1.33E−05), Chr16_6275806 (*P* = 4.14E−06), and Chr16_6275823 (*P* = 6.05E−06), which were significantly associated with HC (Figure 2b). Remarkably, we also found that those three nonsynonymous variants exhibited strong linkage disequilibrium (LD, *r* ^2^ > 0.8) with the 6‐bp InDels in *PtoDPb1* (Figure 2c,d). Based on these LD‐blocks, we classified the 435 *P. tomentosa* accessions into two haplotype groups, Hap1 (haplotype group 1; *n* = 256) and Hap2 (*n* = 157) (MAF > 0.05, Figure 2e). Statistically, the HC value of accessions with Hap1 was significantly higher, by 21.15%, than that of Hap2 (*P* = 3.29E−06, *t*‐test; Figure 2f), and the four nature allelic variations had different genetic effects on HC (Figure 2g). The observed pleiotropic effects and the phenotypic variation suggest that *PtoDPb1* allelic variations might play a vital role in the wood formation process of *Populus*. We therefore postulate that *PtoDPb1* impacts wood formation, possibly via the carbohydrate metabolic pathway.

### Overexpression of *PtoDPb1* improved cellulose content and altered fibre morphology

**Figure 3:**
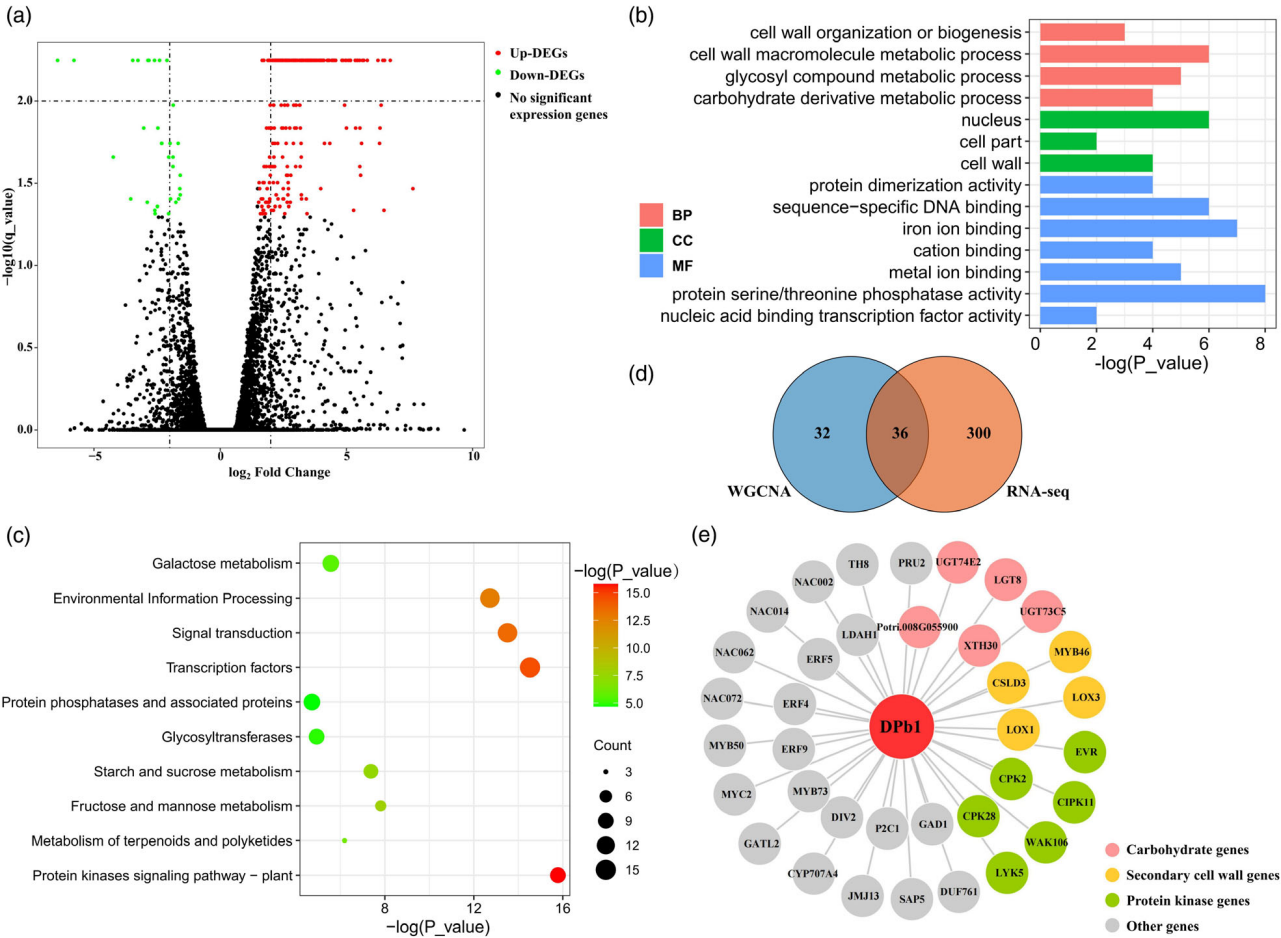
The transcriptional analysis of *PtoDPb1*‐overexpressing (OE) plants. (a) A volcanic plot derived from RNA‐sequencing (RNA‐seq) analysis. Green dots represent down‐regulated differentially expressed genes (DEGs), red dots represent up‐regulated DEGs, and black dots represent genes with no significant change in expression. (b) Gene Ontology (GO) enrichment analysis for 336 DEGs of *PtoDPb1*‐OE plants. The abbreviations BP, CC, and MF correspond to biological process, cellular component, and molecular function, respectively. (c) Kyoto Encyclopedia of Genes and Genomes (KEGG) analysis for 336 DEGs found in *PtoDPb1*‐OE plants. The sphere's size corresponds to the number of genes, while its colour signifies the magnitude of the *P* value. (d) A Venn diagram illustrating the intersecting genes between those co‐expressed with *PtoDPb1* and the DEGs identified from RNA‐seq analysis. (e) The 36 overlapping genes detected through weighted gene co‐expression network analysis (WGCNA) and RNA‐seq analysis. Pink circles represent overlapping genes associated with carbohydrate metabolism. Yellow circles represent overlapping genes related to secondary cell wall formation. Green circles represent overlapping genes related to protein kinase activity. Grey circles denote other overlapping genes.

To functionally characterize the *PtoDPb1* gene, we engineered three independent transgenic *Populus* lines overexpressing *PtoDPb1* (designated *PtoDPb1*‐OE) (Figure 2h and Figure S3a). The reverse transcription real‐time quantitative PCR (RT‐qPCR) analysis revealed that the average transcript levels of *PtoDPb1* in *PtoDPb1*‐OE plants were 7.52‐fold higher than those in their wild‐type (WT) counterparts (*P* < 0.05; Figure S3b). When compared to WT, the plant height of *PtoDPb1*‐OE plants was 58.67–61.40 cm, exhibiting a reduction by 18.13%–26.80% (*P* < 0.05, Figure 2h and Figure S3c), while the stem diameter showed no significant difference in 2‐old‐month transgenic lines (Figure S3d). In assessing fibre morphology, our initial focus was on fibre cell size. Compared to the wild type, *PtoDPb1* overexpression led to a substantial increase of 20.00%–23.33% in the xylem fibre cell layers, with the number of xylem fibre cells per square millimetre expanding by 26.47%–50.00% (*P* < 0.05, Figure 2i–l). Simultaneously, individual xylem fibre cell size decreased within the range of 15.51%–20.95% (*P* < 0.01, Figure 2m), whereas phloem fibre cell size exhibited no significant change compared to WT (Figure S3e–h). Further investigation of fibre shape revealed a reduction in fibre length (ranging from 12.09%–16.24%, *P* < 0.01), accompanied by an increase in fibre width (ranging from 29.49%–44.33%, *P* < 0.01, Figure 3n–p), leading in an overall expansion of fibre dimension (ranging from 13.74%–23.15%, *P* < 0.01, Figure S3i). These findings indicated that the *PtoDPb1* overexpression impacted plant growth and nutrition, consequently resulting in variation in fibre morphology.

Subsequently, to determine whether *PtoDPb1* exerts extensive effects on the formation of secondary cell walls in stems, we conducted a chemical composition analysis. This analysis revealed an increase in lignin content in the stems of transgenic lines by 18.37%–30.04% compared to the WT (*P* < 0.05, Table 1). Corroborating this, histochemical staining demonstrated an enhancement in the lignified cell layers of mature xylem by 42.86%–64.29% in the stems of *PtoDPb1*‐OE plants compared to the WT (*P* < 0.05, Figure S2j). We further assessed monosaccharide contents in the stem and found that glucose content was significantly elevated by 48.68%–60.58% (*P* < 0.01, Table 1), and cellulose content remarkably surged by 47.96%–60.33% (*P* < 0.01, Table 1) in the transgenic lines compared to the WT. Concurrently, the contents of the main monosaccharides constituting hemicellulose, including xylose, arabinose, and galactose, also exhibited alterations in the stems of transgenic plants. Specifically, the contents of xylose and arabinose significantly decreased by 12.57%–16.25% and 21.33%–25.75% (*P* < 0.05, Table 1), respectively, whereas the galactose content increased by 18.75%–22.10% (Table 1). As a result, hemicellulose content decreased by 21.61%–22.90% in *PtoDPb1*‐OE plants compared to the WT (Table 1). Taken together, these findings substantiate the role of *PtoDPb1* in wood formation by modulating *Populus* monosaccharide content and fibre morphology.

**Table 1:** Chemical component analysis in stem of *PtoDPb*1‐OE plants

	WT	OE‐3	OE‐5	OE‐8
Lignin content^†^	18.51 ± 0.010	24.07 ± 0.020*	23.13 ± 0.022*	21.91 ± 0.016*
Cellulose content^†^	25.46 ± 0.001	38.30 ± 0.002**	37.67 ± 0.002*	40.82 ± 0.012**
Hemicellulose content^†^	19.39 ± 0.010	15.59 ± 0.003*	16.22 ± 0.001**	16.16 ± 0.001*
Glucose^‡^	79.23 ± 0.016	119.87 ± 0.017**	117.80 ± 0.059*	127.23 ± 0.071**
Xylose^‡^	58.83 ± 0.004	49.70 ± 0.013*	51.43 ± 0.006**	49.27 ± 0.005*
Arabinose^‡^	25.36 ± 0.019	19.23 ± 0.040*	19.95 ± 0.021*	18.83 ± 0.046*
Galactose^‡^	15.52 ± 0.092	18.43 ± 0.034	18.86 ± 0.055	18.95 ± 0.063
Galacturonic acid^‡^	4.14 ± 0.047	3.99 ± 0.081	3.95 ± 0.027	4.15 ± 0.039

The differences of tested indexes were compared with wide type using *t*‐test, *P < 0.05, **P < 0.01.

† The lignin content and cellulose content in wild‐type and transgenic lines of *Populus* were expressed as percentage cell wall residues.

‡ The monosaccharide compositions in stem of wild‐type and transgenic lines of *Populus* were presented as mean values ± standard deviations (SD) (g/L cell wall residues).

### Transcriptional analysis identified *PtoDPb1* regulatory networks that shape cellulose content in *P. Tomentosa*

To elucidate the genetic regulatory networks of *PtoDPb1* during wood formation, we collected xylem tissue from *PtoDPb1*‐OE and WT plants for RNA‐sequencing (RNA‐seq). In total, we identified 336 significant differentially expressed genes (DEGs) between *PtoDPb1*‐OE and WT plants (|log_2_ Fold Change| > 1, *q* < 0.05). These comprised 291 up‐regulated and 45 down‐regulated genes in *PtoDPb1*‐OE plants (Figure 3a and Dataset S6). The RT‐qPCR results for 10 randomly selected DEGs aligned well with the RNA‐seq data (*P* < 0.05, Figure S4). GO and KEGG analyses revealed these 336 DEGs were significantly enriched in processes related to glycosyl transferase/hydrolase activity and cell wall macromolecule metabolic processes. This suggests that *PtoDPb1* may be involved in the networks governing SCW formation (Figure 3b,c). Interestingly, genes associated with cellulose biosynthesis exhibited differential expression in the *PtoDPb1*‐OE lines. For example, *Populus* orthologous genes directly or indirectly linked with cellulose formation of primary cell wall, such as *CESA8* (Polko and Kieber, 2019), *CSLC12* (Wang *et al*., 2018), *CSLD3* (Yang *et al*., 2020), and *XTH30* (Yan *et al*., 2019), were up‐regulated in *PtoDPb1*‐OE lines (Dataset S6), although their involvement in secondary cell wall formation has not been demonstrated. This trend aligns with the observed increase in cellulose content in the transgenic lines (Table 1).

Based on the candidate co‐expression module related to wood formation, we identified 68 genes interlinked with *PtoDPb1* (Table S3). Among these, 36 genes overlapped with the DEGs in *PtoDPb1*‐OE plants (Figure 3d,e and Table S4), pointing to a high‐priority regulatory network encompassing *PtoDPb1* and these 36 DEGs. A subset of these 36 DEGs are members of the glycosyl hydrolase family. For instance, *Potri.008G192600* (encoding glucosyl transferase family 8, *LGT8*) and *Potri.008G055900* (encoding Beta‐1,3‐Glucan hydrolase like protein) displayed more than a fourfold up‐regulation in *PtoDPb1*‐OE compared to WT plants. UDP‐glucosyltransferase (UGT) genes, such as *Potri.012G048700* (*UGT73C5*) and *Potri.001G389200* (*UGT74E2*), were also notably up‐regulated in *PtoDPb1*‐OE plants, by 4.62‐fold and 3.30‐fold, respectively, compared to WT plants. Collectively, these findings lend support to the hypothesis that *PtoDPb1* plays a vital role in cellulose biosynthesis via glucose metabolism in *Populus*.

### PtoDPb1 regulates *PtoUGT74E2* by forming a complex with PtoE2Fa

**Figure 4:**
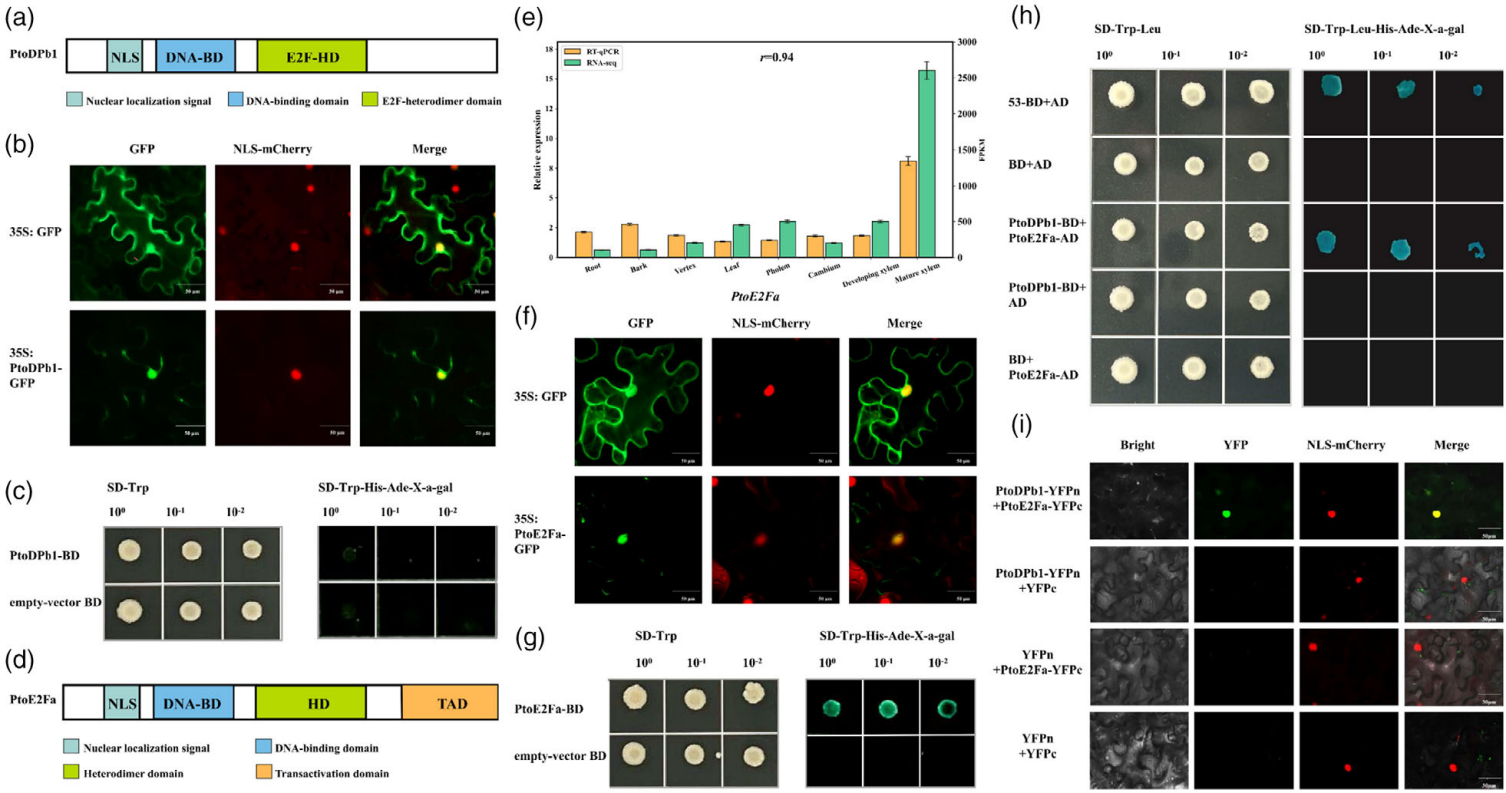
The interaction of PtoDPb1 and PtoE2Fa *in vitro* and *in vivo*. (a) The structural organization of *PtoDPb1*. (b) Subcellular localization of PtoDPb1 using transient expression of 35S:PtoDPb1‐GFP in *Nicotiana benthamiana* leaves. An empty vector serves as a control (*N* = 3). (c) Transcriptional activation analysis of PtoDPb1. (d) The structural organization of *PtoE2Fa*. (e) Tissue‐specific expression analysis of *PtoE2Fa*. The Pearson correlation coefficient (*r*) represents the correlation between *PtoE2Fa* expression levels across different tissues of *Populus tomentosa*, as determined using the reverse transcription real‐time quantitative PCR (RT‐qPCR) and RNA‐sequencing (RNA‐seq). (f) Subcellular localization of PtoE2Fa, demonstrated by transient expression of 35S:PtoDPb1‐GFP in *N. benthamiana* leaves. An empty vector was used as a control (*N* = 3). (g) Transcriptional activation analysis of PtoE2Fa. (h) The interaction between PtoDPb1 and PtoE2Fa in *P. tomentosa* was observed *in vitro* using yeast two‐hybrid (Y2H) assay. Empty vectors, pGADT7 or pGBKT7, served as negative controls (*N* = 3). (i) The *in vivo* interaction between PtoDPb1 and PtoE2Fa was investigated using bimolecular fluorescence complementation (BiFC) assays. A YFP signal was observed in protoplasts of *N. benthamiana* leaves co‐expressing PtoDPb1‐YFPn and PtoE2Fa‐YFPc plasmids. Empty YFPc/empty YFPn, PtoE2Fa‐YFPc/empty YFPn, and PtoDPb1‐YFPn/empty YFPc were used as negative controls (*N* = 3).

Deciphering the downstream genes of *PtoDPb1* for fibre property traits, we executed eQTN mapping between the 389 genetic variants and 28 InDels within *PtoDPb1* and the expression levels of 36 DEGs in *P. tomentosa* mature xylem. This analysis led to the identification of 10 eQTNs and two InDels significantly associated with the expression levels of four distinct genes (*P* ≤ 0.001, *Q* < 0.05, Table S5). To further dissect the downstream gene network of *PtoDPb1*, we observed a significant correlation between the expression levels of *PtoDPb1* and four downstream genes (|*r*| > 0.80) in *P. tomentosa* vascular tissues (Table S5). The PtoDPb1 protein was found to possess a DNA‐binding domain (BD) with a WTTSSCSS (W = A/T, S = G/C) E2F/DP consensus motif (Figure 4a). Intriguingly, promoter analysis revealed two tandem repeats of the E2F/DP‐binding site (TTTGGCCC) within the *PtoUGT74E2* promoter region, specifically between SNP‐1731 and −130. No such binding sites were discernible in the other three downstream genes. Furthermore, the expression correlation between *PtoDPb1* and *PtoUGT74E2* in the mature xylem of six *P. trichocarpa* individuals was strikingly high at 0.901 (Table S5). Reinforcing this observation, Mendelian randomization analysis underscored a positive contribution of *PtoUGT74E2* expression to the HC trait (Table S6 and Figure S5). It is therefore plausible to hypothesize that *PtoUGT74E2* could potentially act as a downstream effector of *PtoDPb1*.

An examination of the amino acid sequence analysis of *PtoDPb1* revealed the presence of a nuclear localization signal, a DNA BD region conforming to the E2F/DP consensus motif, and an HD region, suggestive of its ability to form complexes with E2F members (Figure 4a). Subcellular localization studies indicated that the *PtoDPb1*‐GFP fusion protein was predominantly nuclear (Figure 4b). Interestingly, transcriptional activation analysis suggested that PtoDPb1 itself lacked inherent transcriptional activation potential (Figure 4c). Previous studies have shown that the *Arabidopsis* E2F family member, *AtE2Fa*–*c*, requires heterodimerization with its partner genes, *AtDPs*, for efficient DNA‐binding and the regulation of reporter gene expression (Kosugi and Ohashi, 2002). In *P. tomentosa*, the E2F/DP family comprises four classical E2F members (Figure S6), which necessitate DPs to form functional heterodimers that regulate downstream genes. Tissue‐specific expression profiling revealed that *PtoE2Fa* was predominantly expressed in *P. tomentosa* mature xylem, as assessed by RT‐qPCR (Figure 4d,e and Figure S7). Subsequent subcellular localization and transcriptional activation studies demonstrated that PtoE2Fa displayed transcriptional activation capacity within the nucleus (Figure 4f,g). To ascertain whether the PtoE2Fa‐DPb1 complex was also conserved in *P. tomentosa*, we employed yeast two‐hybrid assay (Y2H) and bimolecular fluorescence complementation (BiFC) studies. These analyses confirmed the interaction between PtoE2Fa and PtoDPb1 (Figure 4h,i). Based on these findings, we postulate that the PtoE2Fa‐PtoDPb1 complex serves as the transcriptional activator to regulate *PtoUGT74E2* expression.

**Figure 5:**
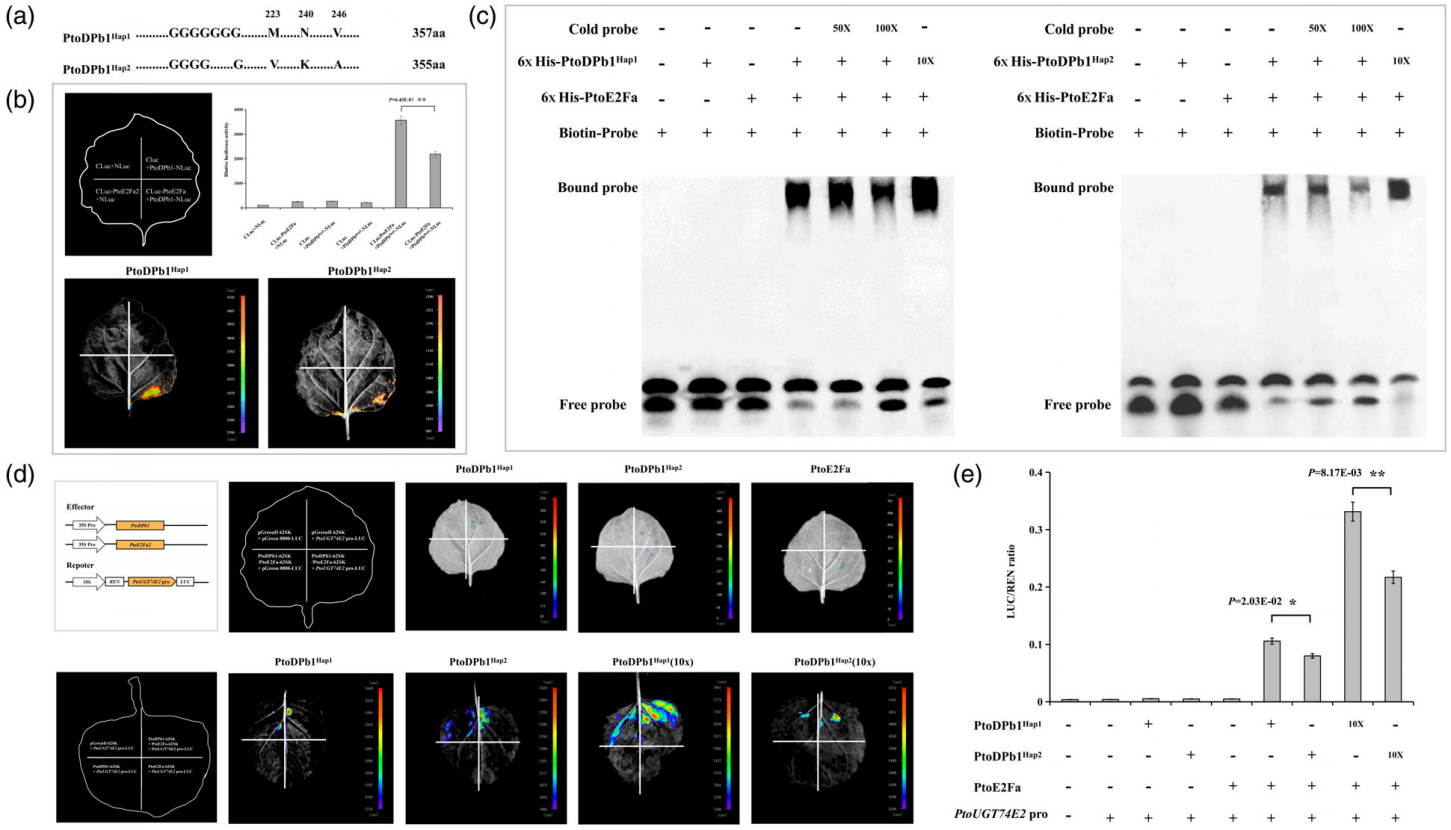
PtoE2Fa‐PtoDPb1 complex activation of *PtoUGT74E2* expression via the WTTSSCSS E2F/DP motif. (a) The amino acid (aa) deletions or substitutions distinguishing PtoDPb1^Hap1^ from PtoDPb1^Hap2^. (b) The in vivo interaction between PtoDPb1^Hap1/Hap2^ and PtoE2Fa *in vivo* was investigated using luciferase bioluminescence imaging (LCI) assay. The firefly luciferase complementation assay in young *Nicotiana benthamiana* leaves. Error bars denote the standard deviation (SD) from three biological replicates. (c) The binding affinity of the PtoE2Fa‐PtoDPb1^Hap1/Hap2^ complex to the promoter of *PtoUGT74E2* by was evaluated through an electrophoretic mobility shift assay (EMSA). The probe sequence, isolated from *PtoUGT74E2*, consists of the WTTSSCSS E2F/DP motif. ‘+’ and ‘−’ indicate the presence and absence of reagents in the lane during protein electrophoresis, respectively. His‐labelled probes with PtoDPb1^Hap1^ and PtoDPb1^Hap2^ proteins are shown. The bound probe represents the relative binding affinity between PtoDPb1^Hap1^/PtoDPb1^Hap2^ alleles and the *PtoUGT74E2* promoter. Protein concentrations were 600 ng/μL. A reciprocal competitive EMSA was performed to evaluate the binding of recombinant PtoDPb1^Hap1^/PtoDPb1^Hap2^ protein to the W‐box motifs using indicated biotin‐labelled probes and unlabelled competitors. For each probe, 50×, and 100×, excess competitor was added. (d, e) The activation regulation of *PtoUGT74E2* by the PtoE2Fa‐PtoDPb1 complex was detected using a dual‐luciferase assay (DLRA). Luciferase/Renilla Luciferase (LUC/REN) ratio was low in *N. benthamiana* leaves expressing *PtoUGT74E2*‐pro‐LUC, but it was strong in those co‐expressing 35S:PtoDPb1, 35S:PtoE2Fa, and *PtoUGT74E2‐*pro‐LUC. The error bars stand for standard deviation (SD) of three biological replicates, and significant differences were determined using *t*‐test, *P < 0.05, **P < 0.01.

In an effort to discern whether the three nonsynonymous variants (A667G, T720G, and T737C mutations) in the HD region and the 6‐bp InDels of *PtoDPb1* influence complex formation with PtoE2Fa (Figure 2e), we performed targeted mutagenesis on the coding sequences of *PtoDPb*1^Hap1^ and *PtoDPb1* ^Hap2^. We removed six nucleotides and substituted the Met^223^ residue with Val, the Asn^240^ residue with Lys, and the Val^246^ residue with Ala (Figure 5a and Figure S2). We employed a luciferase bioluminescence imaging (LCI) assay, which demonstrated that the luciferase activity of the PtoE2Fa‐PtoDPb1^Hap1^ complex was stronger by 85.75% than that of the PtoE2Fa‐PtoDPb1^Hap2^ complex (*P* = 6.43E−03, Figure 5b). These results suggested that these mutations impair heterodimer formation and diminish the interaction efficacy of the PtoE2Fa‐PtoDPb1 complex. To probe further whether allelic variations and 6‐bp InDels in *PtoDPb1* affected the transcriptional activity in binding to the WTTSSCSS E2F/DP motifs of *PtoUGT74E2*, we performed electrophoretic mobility shift assays (EMSA). These assays revealed that the PtoE2Fa‐PtoDPb1^Hap1^ complex demonstrated higher DNA binding affinity than the PtoE2Fa‐PtoDPb1^Hap2^ complex (Figure 5c). The DNA binding affinity strengthened as the concentration of either PtoDPb1^Hap1^ or PtoDPb1^Hap2^ protein was increased, but the binding activity of the PtoE2Fa‐PtoDPb1^Hap1^ complex outperformed that of the PtoE2Fa‐PtoDPb1^Hap2^ complex (Figure 5c).

As anticipated, the dual‐luciferase reporter assay (DLRA) showed that the PtoE2Fa‐PtoDPb1^Hap1^ complex exhibited a 32.27% increase in binding ability to *PtoUGT74E2*, acting as a transcriptional activator, compared to the PtoE2Fa‐PtoDPb1^Hap2^ complex (*P* = 2.03E−02, Figure 5d,e). When the PtoDPb1 protein was augmented 10‐fold, the binding ability of the PtoE2Fa‐PtoDPb1^Hap1^ complex to *PtoUGT74E2* significantly outperformed that of the PtoE2Fa‐PtoDPb1^Hap2^ complex by 42.66% (*P* = 8.17E−03, Figure 5d,e). These data cohered with our previous analysis, which established that the HC of *PtoDPb1* ^Hap1^ was significantly higher by 21.15% compared to that of *PtoDPb1* ^Hap2^. These findings collectively suggested that allelic variations in *PtoDPb1* modulate the regulatory effects on *PtoUGT74E2* by influencing protein interaction efficiency and binding capacity. To validate the impact of *PtoUGT74E2* on fibre traits, we generated transgenic *Populus* plants expressing *PtoUGT74E2* (designated *PtoUGT74E2*‐OE) (Figure S8a–c). Compared to WT, the plant height of *PtoUGT74E2*‐OE plants is in the range of 44.73–44.91 cm, showing a decrease of 20.11%–25.81%, accompanied by increased stem cellulose content (55.10%–63.34%) (Figure S8d,e). Fibre morphology evaluation revealed expanded xylem fibre cell numbers per square millimetre in *PtoUGT74E2*‐OE plants than those in WT, ranging from 19.25%–32.43% (*P* < 0.05, Figure S8f–i). Furthermore, there was a significant decrease in fibre length, ranging from 15.37%–20.24%, along with a slight increase in FW in *PtoUGT74E2*‐OE plants compared to WT (*P* < 0.05, Figure S8k–m). However, individual xylem fibre cell size and fibre dimension within *PtoUGT74E2*‐OE and WT plants remained relatively consistent (Figure S8j,n). Overall, overexpressing *PtoUGT74E2* led to heightened cellulose content and introduced modifications in fibre morphology.

### The allelic variations of *PtoDPb1* affect the interaction efficiency between PtoWAK106 and PtoDPb1

**Figure 6:**
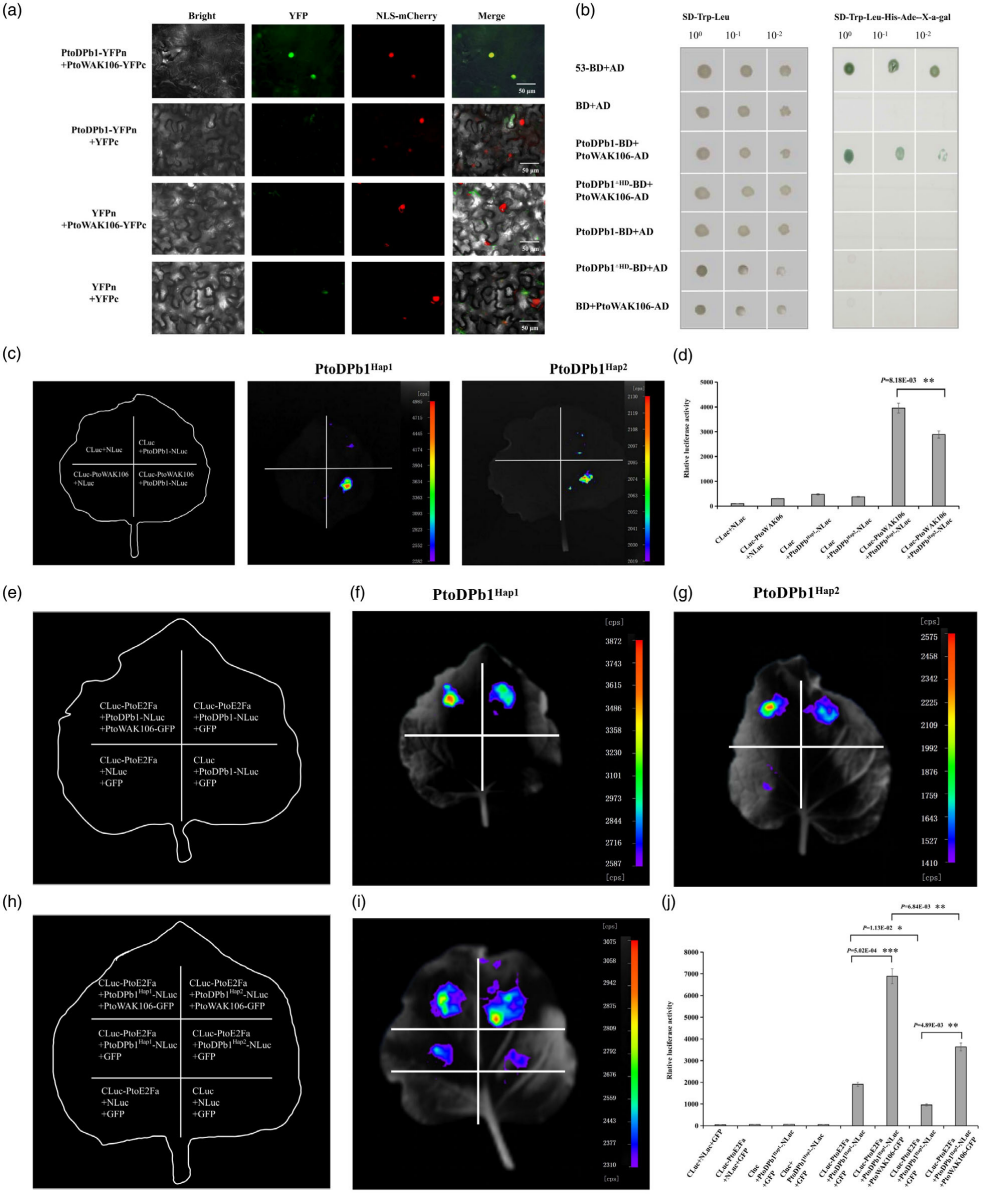
The interaction between PtoDPb1 and PtoWAK106 *in vitro* and *in vivo*. (a) The *in vivo* interaction between PtoDPb1 and PtoWAK106 was tested using bimolecular fluorescence complementation (BiFC) assays. A YFP signal was detected in the protoplasts of *Nicotiana benthamiana* leaves co‐expressing PtoDPb1‐YFPn and PtoWAK106‐YFPc plasmids. Empty YFPc/empty YFPn, PtoWAK106‐YFPc/empty YFPn, and PtoDPb1‐YFPn/empty YFPc served as negative controls (*N* = 3). (b) The *in vitro* interaction between PtoDPb1, PtoDPb1^ΔHD^, and PtoWAK106 in *Populus tomentosa* was detected *in vitro* by yeast two‐hybrid (Y2H) assays. The empty vectors, pGADT7 or pGBKT7, were used as negative controls (*N* = 3). (c, d) The *in vivo* interaction between PtoDPb1^Hap1/Hap2^ and PtoWAK106 was tested using a luciferase bioluminescence imaging (LCI) assay. This firefly luciferase complementation assay was performed in young *N. benthamiana* leaves. (e–j) Impact of protein interaction between the PtoWAK106 and PtoE2Fa‐PtoDPb1 complex. The *in vivo* interaction between PtoDPb1^Hap1/Hap2^‐NLuc, Cluc‐PtoE2Fa, and PtoWAK106‐GFP was tested via a LCI assay. This firefly luciferase complementation assay was performed in young *N. benthamiana* leaves. Error bars stand for standard deviation (SD) of three biological replicates, and significant differences were determined using a *t*‐test, *P < 0.05, **P < 0.01, ***P < 0.001.

To further identify the upstream regulators of *PtoDPb1*, we conducted eQTN mapping for 4473 SNPs within 36 DEGs and *PtoDPb1* expression levels in mature xylem from 435 *P. tomentosa* accessions. We identified nine SNPs associated with five DEGs that significantly correlated with *PtoDPb1* expression levels (*P* ≤ 2.24E−04, employing a Bonferroni correction, *P* = 1/*n*; Figure S9 and Table S7). Subsequent expression correlation analyses between *PtoDPb1* and these five potential regulators in *P. tomentosa* vascular tissues revealed a robust correlation between *PtoWAK106* and *PtoDPb1* (*r* = 0.928, *P* = 6.46E−05). This correlation was reinforced by a high correlation coefficient of 0.903 between the expression of *PtoWAK106* and *PtoDPb1* in the mature xylem of six *P. trichocarpa* individuals (Table S7). These findings led us to hypothesize a potential regulatory network involving *PtoWAK106*‐*PtoDPb1‐PtoE2Fa* and *PtoUGT74E2* for fibre properties. To examine whether PtoWAK106 was a direct upstream regulator of *PtoDPb1*, BiFC analysis confirmed that PtoWAK106 interacted with PtoDPb1 (Figure 6a). To ascertain whether this interaction was dependent on the HD region, we engineered *PtoDPb1* and *PtoDPb1* truncated isomers, which lacked the HD (ΔHD). Y2H demonstrated that the truncated PtoDPb1^ΔHD^ failed to interact with PtoWAK106 (Figure 6b). Subsequent LCI analysis revealed that the allelic variation of *PtoDPb1* attenuated the interaction efficiency between PtoWAK106 and PtoDPb1. The luciferase activity of PtoWAK106‐PtoDPb1^Hap1^ was higher by 37.02% than that of PtoWAK106‐PtoDPb1^Hap2^ (*P* = 8.18E−03, Figure 6c,d).

We next sought to explore the protein interaction effects between the PtoWAK106 and PtoDPb1‐PtoE2Fa complexes. To do this, we expressed PtoWAK106 in the presence of the PtoE2Fa‐PtoDPb1 complex. The luciferase activities of Cluc‐PtoE2Fa/PtoDPb1‐Nluc/PtoWAK106‐GFP samples were found to be significantly higher than those of Cluc‐PtoE2Fa/PtoDPb1‐Nluc/GFP samples. Furthermore, samples containing PtoDPb1^Hap1^ and PtoDPb1^Hap2^ showed a 3.61‐fold and 3.80‐fold increase in luciferase activities, respectively, compared to Cluc‐PtoE2Fa/PtoDPb1^Hap1/Hap2^‐Nluc/GFP samples (*P* < 0.01, Figure 6e–g,j). As predicted, the luciferase intensity in Cluc‐PtoE2Fa/PtoDPb1^Hap1^‐Nluc/PtoWAK106‐GFP samples was dramatically increased by 47.30% compared to the Cluc‐PtoE2Fa/PtoDPb1^Hap2^‐Nluc/PtoWAK106‐GFP samples (*P* = 3.38E−02, Figure 6h–j). Subsequently, we investigated the impact of PtoE2Fa on the protein interactions between PtoWAK106 and PtoDPb1. However, no discernible differences in luciferase activities were observed between Cluc‐PtoWAK106/PtoDPb1‐Nluc/PtoE2Fa‐GFP and Cluc‐PtoWAK106/PtoDPb1‐Nluc/GFP samples (Figure S10). Collectively, these results suggest that PtoWAK106 enhances the physical interactions within the PtoE2Fa‐PtoDPb1 complex.

## Discussion

**Figure 7:**
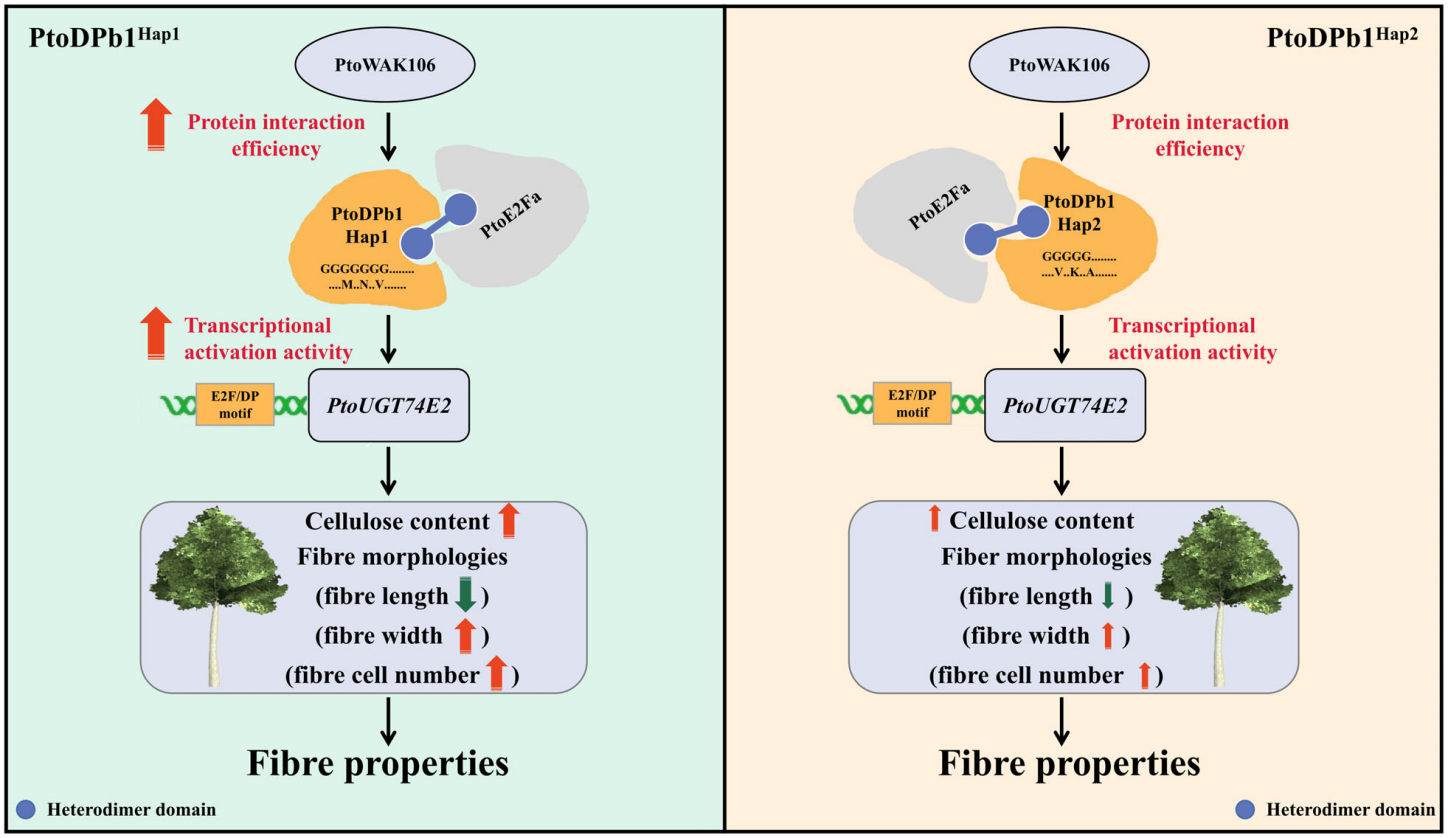
Proposed functional module of *WAK106*‐*DPb1*‐*E2Fa‐UGT74E2* for fibre properties in *Populus tomentosa*. The *PtoDPb1* alleles, positively regulated by *PtoWAK106*, exhibit allelic variations in the coding region (CDS) of *PtoDPb1* that affect protein interaction efficiency and transcriptional activation activity. *PtoWAK106* enhances the stability of PtoE2Fa‐PtoDPb1^Hap1^ heterodimer complex (left). This complex exhibits a stronger binding affinity with the *PtoUGT74E2* promoter, regulating cellulose content and fibre morphologies in *P. tomentosa*. PtoDPb1^Hap2^ (right), in contrast, has lower protein interaction efficiency with PtoWAK106 and PtoE2Fa, and lower transcriptional activation activity of *PtoUGT74E2* than PtoDPb1^Hap1^. Therefore, the *PtoWAK106*‐*PtoDPb1* ^Hap1^‐*PtoE2Fa*‐*PtoUGT74E2* module exhibits superior fibre properties in *Populus*.

Wood formation, a distinctive characteristic of perennial woody plants, is intricately regulated through complex pathways. Identifying the key regulatory factors in wood formation is a crucial step in enhancing tree genetic improvement and breeding. In this study, we unveil *PtoDPb1* as a regulator that induces wood formation in *Populus* by influencing cellulose content and fibre morphology. Specifically, we identified a molecular module, *PtoWAK106*‐*PtoDPb1* ^Hap1^‐*PtoE2Fa*‐*PtoUGT74E2*, which plays a pivotal role in determining fibre properties in *Populus* (Figure 7). This discovery provides a theoretical foundation for using molecular‐assisted breeding to enhance the fine‐fibre properties of forest trees.

### Systems genetics analysis identified a transcriptional regulatory network controlling wood properties in *P. tomentosa*

The transcriptional regulation of wood formation in perennial woody plants, including the accumulation and characteristics of lignocellulosic biomass, is a highly regulated process (Demura and Fukuda, 2006; Mizrachi and Myburg, 2016). Therefore, comprehending the genetic regulatory networks involved in wood formation is essential for improving woody biomass in trees. Currently, the main method for understanding the transcriptional regulatory mechanism of wood formation is reverse genetic analysis of woody tissue genes (Ehlting *et al*., 2005; Fukuda, 2004). While co‐expression analysis is a powerful tool for identifying co‐regulated and functionally related gene modules (Yang *et al*., 2011), it has limitations in revealing the underlying genetic regulatory mechanisms of gene co‐expression networks (Serin *et al*., 2016). However, an intriguing avenue lies in association genetics studies, which establish statistical links between genotypes and phenotypes, enabling the investigation of allelic genetic mechanisms within co‐expression modules. This approach allows for the dissection of functional gene pathways within the co‐expression network (Ingvarsson and Street, 2010). In this study, we aimed to investigate the causative genes influencing wood formation by constructing a co‐expression network of vascular tissues from *P. tomentosa* and *P. trichocarpa*. This network comprised 158 high‐priority core genes exhibiting similar expression patterns, potentially sharing a common regulatory pathway associated with carbohydrate metabolism (Figure 1 and Dataset S5). Through association genetic studies, we explored the genetic effects of the core co‐expression module and made a significant discovery – an association between HC, T6P, G6P, and a key gene, *PtoDPb1*, was identified as a partner gene of E2Fs (Figure 2a). This finding highlights its potential role in modulating carbohydrate metabolism in trees, with implications for cellulose content. Our hypothesis was confirmed by phenotypic measurement of *PtoDPb1*‐OE transgenic plants, which exhibited increased cellulose content and fibre size (Figure 2i–p and Table 1).

By integrating the co‐expression module and DEGs of *PtoDPb1*‐OE transgenic plants, we identified 36 overlapping genes, leading to the speculation of a more refined network involving *PtoDPb1* and these genes, which are implicated in glycosyl transfer and secondary cell wall formation (Figure 3 and Table S4). In addition to co‐expression analysis, we employed the valuable technique of eQTN mapping to unravel the intricate genetic architecture of gene expression regulation and the underlying gene regulatory networks contributing to complex traits (Cubillos *et al*., 2012; Deng *et al*., 2017). Thus, we further identified four downstream genes and five upstream regulators in the *PtoDPb1* regulatory network by eQTN mapping, emphasizing the complexity of this network (Tables S5–S7). Through the integration of co‐expression analysis, association genetics study, and eQTN mapping strategies, we achieved a comprehensive understanding of the regulatory network involving *PtoWAK106*‐*PtoDPb1*‐*PtoUGT74E2* in *Populus*. This integrated approach provided detailed insights into the genetic associations among these genes and shed more light on their potential roles in determining fibre properties.

### PtoE2Fa‐PtoDPb1 complex positively regulated *PtoUGT74E2* affecting fibre properties by interacting with PtoWAK106 in *P. Tomentosa*

TFs, as controllers of transcription initiation, influence such important biological functions as gene regulation and the complexity of SCWs development and differentiation (Du and Groover, 2010; Ruonala *et al*., 2017). It has been observed that partner genes of TFs do not individually activate or repress transcription initiation as monomers; however, dimerizing TFs are at the core of many regulatory circuits that generate the complexity of organisms (Amoutzias *et al*., 2008). For instance, TMO5 forms a heterodimer with LONESOME HIGHWAY (LHW), an atypical bHLH transcription factor, to promote the periclinal division of provascular and procambial cells (De Rybel *et al*., 2013; Ohashi‐Ito *et al*., 2014). In wheat, two specific homologues, *TaDrAp1‐B4* and *TaDrAp2‐B1*, function as partner genes of a transcription repressor (NC2), coordinating plant development and drought tolerance (Zotova *et al*., 2020). In *P. tomentosa*, *PtoDPb1* or *PtoDPb2*, as the E2F/DP family members, form heterodimers with classical E2Fs (Magyar *et al*., 2000). These E2Fs consist of three classical transcriptional activators, namely, *PtoE2Fa*, *PtoE2Fb1*, and *PtoE2Fb2*, which are homologous to *Arabidopsis E2Fa* and *E2Fb* (de Jager *et al*., 2001; Mariconti *et al*., 2002). Additionally, *PtoE2Fc* is a proposed transcriptional repressor homologous to *Arabidopsis E2Fc* (de Jager *et al*., 2001; Mariconti *et al*., 2002). Previous studies have demonstrated that *SKP2a*, a component of the Skp1‐Cul1‐F‐box (SCF) complexes, can bind to and facilitate the degradation of the transcriptional repressor *E2Fc*‐*DPb* complex, allowing for cell cycle progression in *Arabidopsis* (Del Pozo *et al*., 2002, 2006). Notably, Taylor‐Teeples *et al*. (2015) observed a significant increase in crystalline cellulose in the *E2Fc*‐RNAi *Arabidopsis* lines. However, in this study, we revealed the positive regulation of fibre properties by *PtoE2Fa*‐*PtoDPb1* heterodimer complex (Table 1 and Figure 4). Wood fibre properties are a consequence of both physical (fibre morphology) and chemical traits (lignocellulose biomass) that tailor the property requirements for various end products (Ai and Tschirner, 2010; Pirralho *et al*., 2014). Regarding the SCW components, we observed a minor increase in lignin content and a significant increase in cellulose content in *PtoDPb1*‐OE plants than those in WT (Table 1), which were supported by the evaluated expression levels of genes directly or indirectly involved in lignin and cellulose biosynthesis pathways, such as *MYB46*, *LAC5*, *CESA8*, and *CSLC12* (Polko and Kieber, 2019; Taylor‐Teeples *et al*., 2015; Wang *et al*., 2015, 2018). Another possibility for this condition is that the changes in lignin composition reduced the lignocellulosic biomass recalcitrance to enzymatic hydrolysis, thus leading to easily dissociate of cellulose and accessibility of enzymes to cellulose (Zhang *et al*., 2019; Zoghlami and Paës, 2019), and we will investigate this possibility of *PtoDPb1* for enzymatic properties in the future. Similar findings have been reported in studies involving the overexpression of *PtoMYB74* and *GRF11*, which led to elevated levels of cellulose and lignin in *Populus* (Li *et al*., 2018; Tian *et al*., 2022). Additionally, since glucose is the major composition of cellulose, and a positive correlation between glucose and cellulose content was observed in the study, which was consistent with previous study (Yoo *et al*., 2017; Zhang *et al*., 2019; Zoghlami and Paës, 2019). These results indicated that the regulatory pathway of *PtoDPb1* involved in cellulose and lignin biosynthesis and thus affecting the SCW structures and lignocellulosic biomass properties. Morphologically, overexpressing *PtoDPb1* induces significant changes in fibre morphology, including an increase in fibre cell number, a decrease in fibre cell size, a reduction in fibre length, an increase in fibre width, and an overall enlargement of fibre dimension (Figure 2i–p and Figure S3i). This aligns with *PtoDPb1*'s involvement in the G1/S phase of cell division (Magyar *et al*., 2000), which affected cell elongation and expansion, led to alterations in fibre shape and fibre cell size. These findings hold significant implications for advancing the sustainable utilization of lignocellulosic biomass.

*E2Fa* is pivotal in controlling cell division and meristem activity in plants (De Veylder *et al*., 2002). Intriguingly, cell wall genes, including those of the xyloglucan endotransglucosylase family and gly/glucosyl transferase members, were found to be up‐regulated in *AtE2Fa*‐*DPa* transgenic plants compared to WT plants (Berckmans *et al*., 2011; Vlieghe *et al*., 2003). Coincidentally, these family members also appeared among the up‐regulated genes in *PtoDPb1*‐OE plants, such as *XTH30*, *LGT8*, *UGT73C5*, and *UGT74E2* (Table S4). It is known that UGT genes influence fibre cell growth and development by participating in cell wall polysaccharides metabolism in poplar (Aspeborg *et al*., 2005; Lin *et al*., 2016; Read and Bacic, 2002). For example, *PtoUGT74E2* is implicated in glucosinolate biosynthesis (Grubb *et al*., 2014; Saito *et al*., 2013), subsequently affecting the fibre shape and fibre cell number in *PtoUGT74E2*‐OE and *PtoDPb1*‐OE plants (Figure 5 and Figure S8). Overexpression of *UGT74E2* in *Arabidopsis* and *Populus* leads to a more compact, shorter stature, and improved stress tolerance (Figure S8a–c) (Tognetti *et al*., 2010). *Arabidopsis DPb*‐OE plants exhibited a severe dwarf phenotype (Del Pozo *et al*., 2006), a characteristic also observed in the *PtoDPb1*‐OE plants in our study (Figure 2g,h). This short status can be attributed to the increased expression of *PtoUGT74E2* driven by the binding of the *PtoE2Fa‐PtoDPb1* complex (Figure 5).

Additionally, recent studies have underscored the critical role that wall‐associated kinases (WAKs) play in the signalling responses to both environmental and developmental cues (Kohorn and Kohorn, 2012; Wu *et al*., 2020). For example, *Xa4*, which encodes a cell wall‐associated kinase, fortifies the cell wall and enhances resistance to bacterial infections by promoting cellulose synthesis (Hu *et al*., 2017). A plethora of environmental stimuli lead to the expression of WAKs that are involved in plant defence responses (Wu *et al*., 2020). In the current study, we identified *PtoWAK106* as the upstream regulator of *PtoDPb1*. Furthermore, *PtoWAK106* was found to increase the stability of the PtoE2Fa‐PtoDPb1 complex (Figure 6e–j). We also identified the up‐regulation of several drought‐resistance‐related genes, such as *WRKY40*, *NAC72*, and *ERF5*, in *PtoDPb1*‐OE plants (Huang *et al*., 2022; Li *et al*., 2022; Yao *et al*., 2021). It is therefore plausible to hypothesize that the expression of PtoWAK106 is induced by drought, which then modulates the function of *PtoDPb1* and its downstream signalling pathways. This potential mechanism warrants in‐depth investigation and presents an intriguing line of research for the future.

### *PtoDPb1* allelic variations affected protein interaction efficiency and transcriptional activation activity in *P. tomentosa*

It is reported that allelic variation in gene sequences plays a vital role in influencing wood fibre development in forest trees (Du *et al*., 2013; Southerton *et al*., 2010). The present study discovered an LD block encapsulating three non‐synonymous mutations within the HD region alongside 6‐bp InDels in *PtoDPb1* (Figure 2). This LD block segregates 435 *P. tomentosa* natural population specimens into two distinct haplotype groups, with individuals bearing the *PtoDPb1* ^Hap1^ demonstrating higher HC values (an increase of approximately 21.15%) compared to their *PtoDPb1* ^Hap2^ counterparts (Figure 2). The non‐synonymous mutation (Leu to Phe) located in bHLH2 dimer domain suppresses the transcription of *PdCYP79D16* and *PdCYP71AN24* in almonds, consequently sweetening the almond kernel (Sánchez‐Pérez *et al*., 2019). Prior research by Ramirez‐Parra *et al*. (2003) has revealed that both the full‐length DP and its truncated variant DP^ΔBD^ (with the BD region deleted) interact effectively with E2Fs of plant and human origin. This finding suggests the potential influence of the HD on the interaction efficiency between AtE2Fa/b/c and DP proteins in plants. In this context, the current study finds that allelic variation impacts the efficiency of PtoE2Fa‐PtoDPb1 complex formation as well as the transcriptional activation activity of *PtoUGT74E2*. The PtoE2Fa‐PtoDPb1 complex exhibited remarkable binding affinity to the *PtoUGT74E2* promoter when a significant quantity of PtoDPb1 protein was introduced. Intriguingly, the binding efficiency of the PtoE2Fa‐PtoDPb1^Hap1^ complex to *PtoUGT74E2* was higher by 42.66% than that of the PtoE2Fa‐PtoDPb1^Hap2^ complex (Figure 5). Both haplotypes showed an affinity for the target genes, indicating that the formation of the PtoE2Fa‐DPb1 heterodimer also influenced the DNA binding efficiency, which was not solely reliant on the DNA BD region.

The operational mechanism of numerous plant receptor kinases has been comprehensively characterized, typically involving ligand‐mediated homodimerization or heterodimerization of the receptor (Clouse, 2002). Our current research also corroborates these findings, demonstrating an interaction between PtoWAK106 and PtoDPb1 that is dependent on the HD region of *PtoDPb1* (Figure 6a–d). Additionally, we have discovered that the PtoWAK106‐PtoDPb1^Hap1^ protein complex exhibits 37.02% greater interaction efficiency than that of the PtoWAK106‐PtoDPb1^Hap2^ complex (Figure 6). These findings suggest that allelic variations within the HD region not only impact the dimerization activity of PtoE2Fa and PtoDPb1 (Figure 5), but also mediate the interaction efficiency between PtoWAK106 and PtoDPb1. Notably, in the context of the PtoE2Fa‐DPb1 complex, the stability of the PtoWAK106‐PtoE2Fa‐DPb1^Hap1^ complex was found to be higher by 47.30% than that of the PtoWAK106‐PtoE2Fa‐DPb1^Hap2^ complex (Figure 6e–j). The significant impact of these variations on the interaction efficiency and stability of the complexes involving PtoWAK106, PtoE2Fa, and PtoDPb1 underscores their potential as targets for molecular manipulation to enhance fibre properties in forest trees. The clustered regularly interspaced short palindromic repeats (CRISPR)/CRISPR‐associated protein 9 (Cas9) system has emerged as an efficient genome‐editing technology that has been successfully applied across a wide array of plant species (Bortesi and Fischer, 2015). Looking ahead, we anticipate generating knockout transgenic plants of *PtoDPb1*, employing the CRISPR/Cas9 system to directionally mutate allelic variation sites within the HD region in *Populus*. These engineered plants will allow us to observe variations in cellulose content and fibre morphologies, thereby providing ample evidence for the role of the module, composed of *PtoWAK106*‐*PtoDPb1*‐*PtoE2Fa*‐*PtoUGT74E2*, in the regulation of fibre properties.

In summary, in the light of the findings from the association study, co‐expression analysis, and eQTN mapping, we propose a cascaded allele‐specific model of *PtoWAK106*‐*PtoDPb1*‐*PtoE2Fa‐PtoUGT74E2* for wood properties, which regulates cellulose content and fibre morphologies (Figure 7). In this model, natural variation in the HD region of *PtoDPb1* influences the protein interaction efficiency of PtoWAK106‐PtoDPb1 (~37.02%) and PtoE2Fa‐PtoDPb1 (~85.75%), respectively. The PtoE2Fa‐PtoDPb1^Hap1^ complex demonstrates a higher binding affinity (~32.27%) to the *PtoUGT74E2* promoter. In addition, PtoWAK106 enhances the strength of the PtoE2Fa‐PtoDPb1^Hap1/Hap2^ complex. Overall, this module may serve as an optimal molecular tool for the genetic enhancement of wood properties in forest trees.

## Experimental procedures

### Association population and phenotypic data

The association population of *P. tomentosa* used in this study was composed of 435 unrelated individuals. In this study, we measured a total of 22 traits in the selected 435 unrelated individuals, ensuring at least three replicates per genotype. These traits encompassed seven wood property traits, five carbohydrate metabolites (Dataset S7), five lignin metabolites, and five flavonoid metabolites. The detailed 435 unrelated *P. tomentosa* individuals and 22 traits are described in Method S1.

### Weighted gene co‐expression correlation network analysis (WGCNA) and expression analysis

To perform WGCNA, we used a collection of 22 vascular tissue transcriptome datasets from *Populus*, which included 14 groups of *P. trichocarpa* and eight groups of *P. tomentosa* (Table S1). From these 22 vascluar tissue samples, the genes treatment processing o, and WGCNA analysis is described in Method S2.

### SNP‐based association study and phylogenetic tree analysis

SNP‐based association study engaged 17 223 SNPs (minor allele frequency (MAF) >0.05, missing data <0.2) drawn from 158 genes, inclusive of a 2000‐bp promoter, a gene body, and a 500‐bp flanking region. Associations were examined between SNPs and 22 traits. These associations were shown in Method S3. An analysis of the phylogenetic tree of E2F/DP members in *P. tomentosa* is shown in Method S4.

### Construction of the *PtoDPb1* overexpression vector and transformation of *Populus*

The *PtoDPb1* overexpression vector, *pBI121‐GFP*, was assembled by placing the full coding region of *PtoDPb1* under the transcriptional control of the CaMV35S promoter. The primer sequences used to amplify *PtoDPb1* are listed in Table S8. The genetic transformation experiment is described in Method S5.

### The reverse transcription real‐time quantitative PCR (RT‐qPCR)

RT‐qPCR is described in Method S6.

### Determination and analysis of chemical composition in *Populus* stems

Analyses of wood chemical composition, including cellulose, hemicellulose, lignin, glucose, xylose, arabinose, galactose, and galacturonic acid, are performed as described in Method S7.

### Hard plant Safranin O‐fast green staining

The methodology for the histochemical analysis of secondary cell wall (SCW) in stem is elucidated in Method S8.

### Morphological analysis of *Populus* fibres

Morphological analysis of *Populus* fibres is described in Method S9.

### RNA‐sequencing analysis

Simultaneously with the measurements of wood property traits, RNA‐seq analysis was performed on stem tissues derived from *Populus* WT and *PtoDPb1*‐OE lines (#3, #6, and #8). Three individual plants served as biological replicates. RNA‐Seq of transgenic poplar is described in Method S10.

### Expression quantitative trait nucleotide (eQTN) mapping and Mendelian randomization (MR) analysis

The eQTN mapping, which establishes associations between genome‐wide SNPs and expression levels of each trait‐associated gene, was executed using methods identical to those employed for association analysis. For eQTN mapping, a total of 36 DEGs, expressed in more than 80% of individuals, were used (Dataset S8). The eQTN mapping and MR analysis are described in Method S11.

### Subcellular localization and transcription activation assay

The analysis of subcellular and transcription activation is described in Method S12.

### Protein interaction analysis

Protein interaction analysis includes yeast two‐hybrid (Y2H) assay, bimolecular fluorescence complementation (BiFC) analysis, and luciferase complementation imaging (LCI) assay. These experiments are described in Method S13.

### Transcriptional regulation analysis

Transcriptional regulation analysis includes electrophoretic mobility shift assay (EMSA) and dual‐luciferase reporter assay (DLRA). These experiments are described in Method S14.

### Construction of the *PtoUGT74E2* overexpression vector and transformation of *Populus*

Construction of the *PtoUGT74E2* overexpression vector and transformation of *Populus* are followed by Method 4. The primer sequences used to amplify *PtoUGT74E2* are listed in Table S8.

## Accession numbers

The transcriptome expression data (three biological replicates per group) are available in the National Center for Biotechnology Information SRA database under accession numbers PRJNA215447, PRJNA316974, and PRJNA515420. The RNA‐seq data for re‐sequencing of one and 5‐year‐old *Populus tomentosa* clone ‘LM50’ different tissues and the raw data of genome re‐sequencing of 435 *Populus tomentosa* individuals and transgenic *Populus* overexpressing *PtoDPb1* have been deposited in the Genome Sequence Archive (GSA) in the BIG Data Center at Beijing Institute of Genomics (BIG), Chinese Academy of Sciences under the accession numbers of CRA004084, CRA000903, and CRA009669, respectively, that are publicly accessible at http://bigd.big.ac.cn/gsa/.

## Conflict of interest

The authors declare that they have no conflicts of interest.

## Author contributions

D.Z. designed the experiments; D.W., S.Q., W.Q., and Y.J. performed the experiments; D.W., W.Q., M.G., and Y.G collected and analysed the data; D.W., M.Q., Y.F., L.X., J.Z., Q.D., and D.Z. wrote the manuscript; Q.L. and Y.A.E. revised the manuscript, and D.Z. obtained funding and is responsible for this article. All authors read and approved the manuscript.

## Supporting information


**Dataset S1** Gene Ontology (GO) enrichment analysis for each module.
**Dataset S2** Kyoto Encyclopaedia of Genes and Genomes (KEGG) analysis for each module.
**Dataset S3** The 453 high‐connectivity genes from the green module in *Populus tomentosa*.
**Dataset S4** The 220 priority candidate genes from the green module in *Populus tomentosa*.
**Dataset S5** The 158 high‐priority core genes derived from the green module.
**Dataset S6** The 336 different expression genes (DEGs) identified using RNA‐sequencing (RNA‐seq) analysis.
**Dataset S7** Five carbohydrate metabolites contents of 435 unrelated individuals in *Populus tomentosa*.
**Dataset S8** Gene expression data from mature xylem used for expression quantitative trait nucleotide (eQTN) mapping.

**Figure S1** Tissue‐specific expression analysis of *PtoDPb1*.
**Figure S2** Sequence alignment of PtoDPb1^Hap1^ and PtoDPb1^Hap2^ proteins.
**Figure S3** The phenotypes of *PtoDPb1*‐overexpressing lines.
**Figure S4** Correlation analysis of expression levels of 10 randomly selected differentially expressed genes by the reverse transcription real‐time quantitative PCR (RT‐qPCR) and RNA‐sequencing (RNA‐seq).
**Figure S5** Estimates of the genetic effects of allelic SNPs in *PtoDPb1* on the expression of *PtoUGT74E2* and HC traits.
**Figure S6** Phylogenetic tree analysis of E2F members and DP members.
**Figure S7** Tissue‐specific expression analysis of *PtoE2Fb1*, *PtoE2Fb2*, and *PtoE2Fc*.
**Figure S8** The phenotypes of *PtoUGT74E2*‐overexpressing lines.
**Figure S9** Significantly associated significant single nucleotide polymorphisms (SNPs) of upstream regulators identified using expression quantitative trait nucleotide (eQTN) mapping.
**Figure S10** Impact of protein interaction between the PtoE2Fa and PtoWAK106‐PtoDPb1.
**Table S1** Transcription profiling of RNA‐seq datasets used in co‐expression analysis.
**Table S2** Details of significant single nucleotide polymorphisms (SNPs) associated with wood property traits and carbohydrate metabolite traits in the association population of *Populus tomentosa*.
**Table S3** The 68 connected genes with *PtoDPb1* using weighted gene co‐expression network analysis (WGCNA).
**Table S4** The 36 overlapping genes detected using weighted gene co‐expression network analysis (WGCNA) and RNA‐sequencing (RNA‐seq) analysis.
**Table S5** Downstream genes identified using expression quantitative trait nucleotide (eQTN) mapping.
**Table S6** Mendelian randomization (MR) results of the relationship of allelic SNPs of *PtoDPb1*, expression of *PtoUGT74E2*, and HC traits.
**Table S7** Upstream regulators identified using expression quantitative trait nucleotide (eQTN) mapping.
**Table S8** The oligonucleotide sequences of primers used in this study.
**Method S1** Association population and phenotypic data.
**Method S2** Weighted gene co‐expression network analysis (WGCNA).
**Method S3** Single nucleotide polymorphism (SNP)‐based association study.
**Method S4** Phylogenetic tree analysis of E2F/DP members.
**Method S5** Genetic transformation of *PtoDPb1* in *Populus*.
**Method S6** The reverse transcription real‐time quantitative PCR (RT‐qPCR).
**Method S7** Determination and analysis of chemical composition in *Populus* stems.
**Method S8** Hard plant Safranin O‐Fast Green staining.
**Method S9** Morphological analysis of *Populus* fibres.
**Method S10** RNA‐sequencing (RNA‐seq) analysis.
**Method S11** Expression quantitative trait nucleotide (eQTN) mapping and Mendelian Randomization (MR) analysis.
**Method S12** Subcellular localization and transcription activation assay.
**Method S13** Protein interaction analysis.
**Method S14** Transcriptional regulation analysis.

## Data Availability

The transcriptome expression data (three biological replicates per group) are available in the National Center for Biotechnology Information SRA database under accession numbers PRJNA215447, PRJNA316974, and PRJNA515420. The RNA‐seq data for re‐sequencing of one and five‐year‐old Populus tomentosa clone ‘LM50' different tissues and the raw data of genome resequencing of 435 Populus tomentosa individuals and transgenic Populus overexpressing PtoDPb1 have been deposited in the Genome Sequence Archive (GSA) in the BIG Data Center at Beijing Institute of Genomics (BIG), Chinese Academy of Sciences under the accession numbers of CRA004084, CRA000903 and CRA009669, respectively, that are publicly accessible at http://bigd.big.ac.cn/gsa/.
