# Supplementary material for: Allelic variations of WAK106‐E2Fa‐DPb1‐UGT74E2 module regulate fibre properties in Populus tomentosa
Source: Plant Biotechnol J. 2023 Nov 21;22(4):970–86. doi: 10.1111/pbi.14239 (PMC10955495; doi:10.1111/pbi.14239)
Supplement: Supplementary file 2 — Figure S1 Tissue‐specific expression analysis of PtoDPb1. Figure S2 Sequence alignment of PtoDPb1Hap1 and PtoDPb1Hap2 proteins. Figure S3 The phenotypes of PtoDPb1‐overexpressing lines. Figure S4 Correlation analysis of expression levels of 10 randomly selected differentially expressed genes by the reverse transcription real‐time quantitative PCR (RT‐qPCR) and RNA‐sequencing (RNA‐seq). Figure S5 Estimates of the genetic effects of allelic SNPs in PtoDPb1 on the expression of PtoUGT74E2 and HC traits. Figure S6 Phylogenetic tree analysis of E2F members and DP members. Figure S7 Tissue‐specific expression analysis of PtoE2Fb1, PtoE2Fb2, and PtoE2Fc. Figure S8 The phenotypes of PtoUGT74E2‐overexpressing lines. Figure S9 Significantly associated significant single nucleotide polymorphisms (SNPs) of upstream regulators identified using expression quantitative trait nucleotide (eQTN) mapping. Figure S10 Impact of protein interaction between the PtoE2Fa and PtoWAK106‐PtoDPb1. Table S1 Transcription profiling of RNA‐seq datasets used in co‐expression analysis. Table S2 Details of significant single nucleotide polymorphisms (SNPs) associated with wood property traits and carbohydrate metabolite traits in the association population of Populus tomentosa. Table S3 The 68 connected genes with PtoDPb1 using weighted gene co‐expression network analysis (WGCNA). Table S4 The 36 overlapping genes detected using weighted gene co‐expression network analysis (WGCNA) and RNA‐sequencing (RNA‐seq) analysis. Table S5 Downstream genes identified using expression quantitative trait nucleotide (eQTN) mapping. Table S6 Mendelian randomization (MR) results of the relationship of allelic SNPs of PtoDPb1, expression of PtoUGT74E2, and HC traits. Table S7 Upstream regulators identified using expression quantitative trait nucleotide (eQTN) mapping. Table S8 The oligonucleotide sequences of primers used in this study. Method S1 Association population and phenotypic data. Method S2 Weighted gene co‐e [file PBI-22-970-s002.zip › pbi14239-sup-0002-Supporting Information.pdf]

The Plant Biotechnology Journal **Supporting Information**

Article title:

Allelic variations of *WAK106-E2Fa-DPb1-UGT74E2* module regulate fiber properties in *Populus tomentosa*

Authors: Dan Wang#, Mingyang Quan#, Shitong Qin#, Yuanyuan Fang, Liang Xiao, Weina Qi, Yongsen Jiang, Jiaxuan Zhou, Mingyue Gu, Yicen Guan, Qingzhang Du, Qing Liu, Yousry A. El-Kassaby and Deqiang Zhang*

The following Supporting Information is available for this article:

Supporting Methods

Method S1 Association population and phenotypic data

Method S2 Weighted gene co-expression network analysis (WGCNA) analysis

Method S3 Single nucleotide polymorphism (SNP)-based association study

Method S4 Phylogenetic tree analysis of E2F/DP members

Method S5 Genetic transformation of *PtoDPb1* in *Populus*

Method S6 The reverse transcription real-time quantitative PCR (RT-qPCR)

Method S7 Determination and analysis of chemical composition in *Populus* stems

Method S8 Hard plant Safranin O-Fast Green staining

Method S9 Morphological analysis of *Populus* fibers

Method S10 RNA-sequencing (RNA-seq) analysis

Method S11 Expression quantitative trait nucleotide (eQTN) mapping and Mendelian Randomization (MR) analysis

Method S12 Subcellular localization and transcription activation assay

Method S13 Protein interaction analysis

Method S14 Transcriptional regulation analysis

Supporting Figures

Figure S1. Tissue specific expression analysis of *PtoDPb1*.

Figure S2. Sequence alignment of PtoDPb1^{Hap1} and PtoDPb1^{Hap2} proteins.

Figure S3. The phenotypes of *PtoDPb1*-overexpressing lines.

Figure S4. Correlation analysis of expression levels of ten randomly selected differentially expressed genes by the reverse transcription real-time quantitative PCR (RT-qPCR) and RNA-sequencing (RNA-seq).

Figure S5. Estimates of the genetic effects of allelic SNPs in *PtoDPb1* on the expression of *PtoUGT74E2* and HC traits.

Figure S6. Phylogenetic tree analysis of E2F members and DP members.

Figure S7. Tissue specific expression analysis of *PtoE2Fb1*, *PtoE2Fb2*, and *PtoE2Fc*.

Figure S8. The phenotypes of *PtoUGT74E2*-overexpressing lines.

Figure S9. Significantly associated significant single nucleotide polymorphisms (SNPs) of up-stream regulators identified using Expression quantitative trait nucleotide (eQTN) mapping.

Figure S10. Impact of protein interaction between the PtoE2Fa and PtoWAK106-PtoDPb1.

Supporting Tables

Table S1. Transcription profiling of RNA-seq datasets used in co-expression analysis.

Table S2. Details of significant single nucleotide polymorphisms (SNPs) associated with wood property traits and carbohydrate metabolite traits in the association population of *Populus tomentosa*.

Table S3. The 68 connected genes with *PtoDPb1* using weighted gene co-expression network analysis (WGCNA).

Table S4. The 36 overlapping genes detected using weighted gene co-expression network analysis (WGCNA) and RNA-sequencing (RNA-seq) analysis.

Table S5. Down-stream genes identified using expression quantitative trait nucleotide (eQTN) mapping.

Table S6. Mendelian Randomization (MR) results of the relationship of allelic SNPs of *PtoDPb1*, expression of *PtoUGT74E2* and HC traits.

Table S7. Up-stream regulators identified using expression quantitative trait

nucleotide (eQTN) mapping.

Table S8. The oligonucleotide sequences of primers used in this study.

Supporting Data

Dataset S1-S8 were provided separately as an Excel file.

Dataset S1. GeneOntology (GO) enrichment analysis for each module.

Dataset S2. Kyoto Encyclopaedia of Genes and Genomes (KEGG) analysis for each module.

Dataset S3. The 453 high-connectivity genes from the green module in *Populus tomentosa*.

Dataset S4. The 220 priority candidate genes from the green module in *Populus tomentosa*.

Dataset S5. The 158 high-priority core genes derived from the green module.

Dataset S6. The 336 different expression genes (DEGs) identified using RNA-sequencing (RNA-seq) analysis.

Dataset S7. Five carbohydrate metabolites contents of 435 unrelated individuals in *Populus tomentosa.*

Dataset S8. Gene expression data from mature xylem used for expression quantitative trait nucleotide (eQTN) mapping.

Supporting Methods

Method S1 Association population and phenotypic data

Association population

The 435 unrelated *P. tomentosa* individuals were randomly selected from 1,047 naturally distributed individuals, thereby representing, to the greatest extent, the natural distribution of *P. tomentosa* across China (30-40°N, 105-125°E). A germplasm bank of *P. tomentosa* was established in 1982 using a randomized complete block design approach to create three clonal replicates. This bank is located in Guanxian County, Shandong Province, China (36° 23'N, 115° 47'E) (Du *et al*., 2012).

Phenotypic data

The wood property traits measured included fiber length (FL, %), fiber width (FW, %), holocellulose content (HC, %), α-cellulose content (CC; %), lignin content (LC; %), hemicellulose content (HEC; %), and microfibril angle (MFA). The carbohydrate metabolites analyzed were Trehalose 6-phosphate (T6P), Glucose 6-phosphate (G6P), D-Fructose 6-phosphate (F6P), DL-Arabinose (DLA), and L-Fucose (LF). Lignin metabolites were caffeic aldehyde (Caa), p-coumar aldehyde (pCoa), L-phenylalanine (LPh), cinnamic acid (Cia), and coniferyl aldehyde (Coa). flavonoid metabolites were apigenin (Ap), butin (Bu), pinocembrin (Pi), naringenin (Na), and eriodictyol (Er). The sampling and measurement methods for these wood property traits, lignin metabolites, and flavonoid metabolites were conducted following the protocols described in previous studies (Du *et al*., 2012; Xiao *et al*., 2019; Lu *et al*., 2020). Carbohydrate metabolites were measured using the method reported by Sluiter *et al*. (2008), with at least three replications per genotype.

Method S2 Weighted gene co-expression network analysis (WGCNA) analysis

The transcriptome data of 22 groups of poplar vascular tissues were used for WGCNA analysis. For *P. trichocarpa*, the vascular tissue data incorporated the cambium described by Liu *et al.* (2014) and the xylem and phloem outlined by Liu *et al.* (2021) and Zinkgraf *et al.* (2017). Various tissue samples, including phloem, cambium, developing xylem, and mature xylem, root, bark, vertex, and leaf, were collected from the *P. tomentosa* clone 'LM50' of both one-year-old and five-year-old clones planted in Guan Xian County. From these tissue samples, total RNA was extracted using the Qiagen RNAeasy kit (Qiagen, Hilden, Germany), following the manufacturer's instructions. Three individuals were used as biological replicates.

WGCNA analysis was based on the expression levels of 24,546 genes. Those genes were selected based on their elevated expression in at least 80% of the vascular tissue under investigation in the *P. tomentosa* clone 'LM50', and exhibited a high FPKM (fragments per kilobase of transcript per million fragments) value, exceeding five. The software tools Cuffquant and Cuffnorm (Trapnell *et al.*, 2012) were utilized to compute these FPKM values. To sculpt the co-expression networks, Log_2-normalized FPKM values were harnessed using the WGCNA (RRID: SCR_003302) package in R (Langfelder and Horvath, 2008, 2012). Following tutorials for WGCNA, we selected a soft thresholding power of 20 to define the adjacency matrix based on the criterion of approximate scale-free topology, with a minimum module size of 100 and a module detection sensitivity of deep split 4 (Zhao *et al.*, 2021). Hub genes within each module were determined by their high correlation in the candidate modules. Genes whose connectivity ranked in the top 10% and weighed more than 0.2 were considered to possess high connectivity (Liu *et al.*, 2019). To identify the modules embedded in the core wood formation pathway, GO and KEGG enrichment analyses were carried out on the expressed genes using TBtools (Chen *et al.*, 2020).

Method S3 Single nucleotide polymorphism (SNP)-based association study

SNP-based association study was analyzed using the Tassel v5.0 software, employing a mixed linear model (MLM) that incorporated kinship coefficients (K) and population structure (Q) as controlling factors (Bradbury *et al.*, 2007). The Q and K matrices were procured in accordance with the method described by Du *et al.* (2018). Normalization of transcript expression was achieved using FPKM. Phenotypic data were further normalized using the $\log_{10}$ (1 + FPKM) transformation (Lu *et al.*, 2020). The threshold for the significance of single SNP-based association results was set at $P \leq$ 5.81E-05 (P = 1 / n where n denotes the number of independent markers, *i.e.,* 17,223 SNPs, which is a rough approximation of the Bonferroni correction).

Methods S4 Phylogenetic tree analysis of E2F/DP members

We aligned protein sequences of the 8 E2F/DP members of *Populus tomentosa* with the ClustalW programme, and constructed phylogenetic trees using the MEGA 6.0 software with the maximum-likelihood method. We used 1,000 bootstrap test replicates and the Poisson model (Tamura *et al*., 2013). 8 *Arabidopsis,* 8 P*opulus trichocarpa* and 8 *Populus euphratica* E2F/DP proteins were also included as reference proteins.

Methods S5 Genetic transformation of *PtoDPb1* in *Populus*

Hybrid poplar tree (*Populus alba* × *Populus glandulosa*) clone 84K was used as the donor plant for transformation. The plants were maintained in a controlled growth chamber with an ambient temperature of 23 - 25 °C, under a 16/8 light/dark cycle with a light intensity of 50 μM/m^2/s provided by cool white fluorescent tubes. 84K leaf discs were infected with a culture (OD_{600} = 0.8) of *Agrobacterium tumefaciens* strain GV3101 harboring the *PtoDPb1*-pBI121:GFP construct. Post-infection, the leaf discs were transferred onto the shoot induction medium (SIM), comprising Murashige-Skoog basal medium laced with 0.5 mg/L 6-benzyl amino purine and 0.05 mg/L naphthalene acetic acid, for a period of five days at 22 ± 2 °C in the dark. The selection of transgenic poplar leaf discs occurred on SIM, supplemented with 50 mg/L Kanamycin and 300 mg/L Timentin, under a 16/8 light/dark regime. Following a month, individual regenerated shoots were detached and transferred to a root induction medium (RIM), consisting of 1/2 Murashige-Skoog medium supplemented with 0.05/L mg-indole-3-butyric acid, 0.05 mg/L naphthalene acetic acid, 50 mg/L Kanamycin, and 300 mg/L Timentin, for adventitious root induction.

All plants underwent propagation via subculturing and were subsequently transferred to soil for phenotype analysis through sectioning. The data compilation involved at least three transgenic lines demonstrating a stable, common phenotype. Measurements of the samples’ stem heights and diameters were taken 60 days after transplantation.

Methods S6 The reverse transcription real-time quantitative PCR (RT-qPCR)

The total RNA was quantified utilizing a NanoDrop spectrophotometer (Thermo Fisher Scientific, Waltham, MA), while RNA integrity was substantiated through agarose gel electrophoresis. RT-qPCR was carried out on a 7500 Fast Real-time PCR system using SYBR Premix Ex Taq (Thermo Fisher Scientific), adhering to the PCR program as reported by Quan *et al.* (2021). The gene-specific primers used for RT-qPCR are listed in Table S8. All reactions were conducted in triplicate, both technically and biologically. *Actin* (EF145577) and 18S ribosomal RNA (18S rRNA) were used as internal controls. The application of the melting curve was instrumental in inspecting the specificity of amplification, and the $2^{-\Delta\Delta Ct}$ method facilitated the analysis of the relative transcript levels of candidate genes (Quan *et al.*, 2021).

Methods S7 Determination and analysis of chemical composition in *Populus* stems

Samples of the entire stem were harvested from two-month-old *PtoDPb*1-OE and WT plants, subsequently ground to a fine powder to yield dried cell wall residue (CWR) at 105 °C for a duration of 4 hours (h) (Meyer *et al.,* 1998). Acid-insoluble lignin content was determined using 0.15 g fresh weight from stem tissues, ground in liquid nitrogen to a fine powder, following the Klason procedure (Lu *et al*., 2013). The alcohol-insoluble residue (AIR) was prepared as previously described (Taylor-Teeples *et al*., 2015), and analyzed using high performance liquid chromatography (HPLC). The analysis of carbohydrate contents was executed based on the protocols outlined by the National Renewable Energy Laboratory (NREL) (Sluiter *et al*., 2008). The neutral sugars during the saccharification process were analyzed using a Waters 2695e HPLC fitted with an Aminex HPX-87P (300×7.8 mm) column (Bio-Rad, Hercules, CA) at 85 °C, and a refractive index detection detector set at 35 °C. The injection volume for the sample was 10 μL, and distilled water was used as the eluent at a flow rate of 0.6 mL/min. The glucose yield was calculated with the assumption that 1 g of cellulose present in the solution theoretically yields 1.11 g of glucose. Assays were conducted in triplicate, with the mean values being subsequently calculated.

Methods S8 Hard plant Safranin O-Fast Green staining

To examine the lignified cell wall in stems, approximately 15th stem internodes of 2 months old transgenic and WT *Populus* were fixed in FAA buffer (50% ethanol, 5% acetic acid, and 3.7% formaldehyde) for 10 days and embedded in paraffin. Three individuals of each transgenic line and WT were used for the histochemical assay. The resin-embedded stem internodes of 2 months old plants were sectioned into 50-80 nm with an ultra-thin slicer and the slices were scooped with a carbon-free aromatic membrane copper net. Firstly, stem internodes were removed from the embedding agent, and rehydration included the following four steps. The sections were put into ethylene glycol ethyl ether acetate I 6 h at 37 °C and ethylene ether acetate II overnight at 37 °C. They were put into ethylene glycol ethyl ether acetate III 10-15 min minutes at room temperature and ethylene glycol ethyl ether acetate IV 10-15 min minutes at room temperature. The sections were rehydrated in 100% - 100% - 95% - 90% - 80% alcohol. Each step takes anywhere from 10 minutes. Lastly, rinse with running water. Then the sections were put into safranin O staining solution for 15-30 s and three cylinders of anhydrous ethanol. Next, the sections were put into three cylinders of 50%, 70%, and 80% alcohol respectively for 3-8 s. The concentrations of alcohol were 50,70 and 80 percent. In addition, sections were put into a fast green staining solution for 4-6 s and three cylinders of anhydrous ethanol for 5 s in turn. Finally, the sections were put into three cylinders of xylene for 5 min, mounted with neutral balsam, and observed under a biological microscope (Nikon ECLIPSE Ci, Japan).

Methods S9 Morphological analysis of *Populus* fibers

A range of morphological parameters of the *Populus* fibers, including the size of the transverse, radial longitudinal, and tangential longitudinal, were measured using a micrometer with a resolution of 0.001 cm. The wood block was bisected vertically to match the stem size, subsequently boiled, vented, and macerated in a test tube containing a 1:1 (v/v) mixture of acetic acid and hydrogen peroxide at 60 °C for 24 h, until the sample turned white and the fibers were entirely dissociated. The samples were then washed three times with distilled water, and the test tube was filled with distilled water to the point of saturation. Vigorous shaking of the test tube ensured a comprehensive dispersion of the wood fibers. A droplet of the cane dye solution was added, followed by the extraction of a small amount of fiber. The length and width of the fibers were then measured under an Olympus BX-31 light microscope (Olympus, Tokyo, Japan). For each specimen, more than 200 fibers were measured (Huang *et al.*, 2022).

Methods S10 RNA-sequencing (RNA-seq) analysis

Library construction and sequencing were undertaken by NovoGene Co., Ltd (Beijing, China). Transcript expression normalization was achieved using the FPKM method. The procedures for transcriptome data processing have been detailed by Quan *et al.* (2019). Different expression genes (DEGs) were identified using the Cuffdiff software and selected for downstream analysis by filtering for $|\log_2 \text{Fold Change}| > 1$ and $q < 0.05$.

Methods S11 Expression quantitative trait nucleotide (eQTN) mapping and Mendelian Randomization (MR) analysis

Expression levels of genes in mature xylem were determined through RNA-seq in an association population of *P. tomentosa*, following the methodology described by Quan *et al.* (2021). Each genotype was represented by three clones serving as biological replicates. RNA library construction and sequencing were performed by NovoGene Co., Ltd. (Beijing, China). The expression levels of trait-related genes were represented by $\log_{10}$ (1 + FPKM) (Xiao *et al.*, 2019). The significance level for association results of SNPs of *PtoDPb1* and expression levels of the 36 different expression genes (DEGs) was set at $P < 0.001$, $Q < 0.05$ to control the false-positive error rate. A total of 4,473 SNPs (MAF > 5%, missing < 20%) were identified from the 36 DEGs (including a 2,000-bp promoter, gene body, and 500-bp flanking region). To control the false-positive error rate, the significance level of the association results of these 4,473 SNPs and the expression level of *PtoDPb1* was set at $P \leq 2.24\text{E-}04$ ($P = 1 / n$; n represented the independent marker number, *i.e.* 4,473 SNPs, which is roughly a Bonferroni correction).

Mendelian Randomization (MR) analysis was conducted to assess the causal connections between genetic variants and traits related to wood characteristics. Genetic markers (SNPs) that displayed significant associations with these wood traits were employed in the MR analysis. Within this study, the MendelianRandomization package in R was applied for the MR analysis, utilizing the inverse-variance weighting (IVW) technique to consolidate the impacts of multiple SNPs, following the approach described by Yavorska and Burgess (2017).

Methods S12 Subcellular localization and transcription activation assay

The analysis of subcellular localization of *PtoDPb1*, *PtoDPb1* was then cloned into the *pBI121* vector, generating a *PtoDPb1*-GFP fusion construct driven by the *35S* promoter (*35S*: *PtoDPb1*-GFP). This construct was introduced into the *A. tumefaciens* strain GV3101, and then transiently expressed in *Nicotiana benthamiana* leaves by infiltration. Two days after the infiltration, fluorescence signals were observed using a Leica SP8 confocal laser scanning microscope (Leica Biosystems, Nussloch GmbH, Germany). Each experiment was performed with three biological replicates.

For the transcription activation assay, the full-length open reading frames (ORFs) of *PtoDPb1* and *PtoE2Fa* were amplified using gene-specific primers. The amplified products were inserted into *pGBKT7* (Clontech, Mountain View, CA), and the resulting recombinant plasmid was introduced into the Y2H yeast strain. The Matchmaker GAL4-based Two-Hybrid system 3 (Clontech) was used for the transactivation activity assay. Each yeast liquid culture was diluted to an absorbance of 0.5 at OD_{600}, and 3 μL of each dilution was inoculated onto the synthetic dextrose (SD) medium lacking tryptophan (SD-Trp) for the selection of positive clones. These were then placed on the SD medium lacking Trp, histidine, and adenine (SD-Trp-His-Ade) for the transactivation assay (Zhao *et al.*, 2018). X-α-gal was used to identify the transcription activation activity of PtoDPb1 and PtoE2Fa. Three biological replicates were carried out for each experiment.

Method S13 Protein interaction analysis

Yeast two-hybrid (Y2H) assay

The full-length coding sequences of *PtoE2Fa* and *PtoWAK106* were fused with the GAL4 activation domain in the prey vector *pGADT7* (Clontech). The truncated coding sequence of *PtoDPb1* was fused with the GAL4 binding domain in the prey vector *pGBKT7* (Clontech). The primer sequences are listed in Table S8. The pairs of constructs, including PtoE2Fa-AD and PtoDPb1-BD, PtoWAK106-AD and PtoDPb1-BD, PtoWAK106-AD and PtoDPb1$^{\triangle HD}$-BD, were co-transformed into Y2H yeast strains and grown on an SD-Leu-Trp medium for 2-3 days. These were then selected on a medium lacking Trp, leucine, His, Ade, and X-α-gal (SD-Trp-Leu-His-Ade-X-a-gal) for 3-5 days to examine the interactions between PtoDPb1 and PtoE2Fa protein. Pairs of empty vectors pGBKT7 and pGADT7 were used as negative controls, while the pGBKT7-p53 and pGADT7-T pairs served as positive controls for protein-protein interaction. Each experiment was performed in triplicate.

Biomolecular fluorescence complementation (BiFC) analysis

The coding sequences of *PtoDPb1* were cloned into the *pSPYNE(R)173* vector. The coding sequences of *PtoE2Fa* and *PtoWAK106* were cloned into the *pSPYCE(MR)* vector. The constructed plasmids were introduced into *A. tumefaciens* GV3101 competent cells. *PtoDPb1/PtoE2Fa* and *PtoDPb1/PtoWAK106* pairs were selected and mixed to infiltrate *N. benthamiana* leaves. After 48 h, the YFP fluorescence signal was observed under a Leica SP8 confocal laser scanning microscope (Leica Biosystems, Nussloch GmbH, Germany), with an excitation wavelength of 488 nm and detection at 499 - 535 nm. The excitation wavelength and detection wavelengths for the nuclear localization signal-mCherry (NLS-mCherry) were set to 587 nm and 600-630 nm, respectively. Each experiment was performed in triplicate.

Luciferase complementation imaging (LCI) assay

A firefly luciferase complementation assay was conducted by infiltrating *N. benthamiana* leaves, according to a previous report (Zhou *et al.*, 2015). The full coding regions of *PtoDPb1*^{Hap1} and *PtoDPb1*^{Hap2} were inserted into the binary vector *pCambia1300-NLuc* to generate *PtoDPb1*^{Hap1}-NLuc and *PtoDPb1*^{Hap2}-NLuc, while the full coding regions of *PtoE2Fa* and *PtoWAK106* were inserted into *pCambia1300-CLuc* to generate CLuc-*PtoE2Fa* and CLuc-*PtoWAK106*. *Agrobacteria* cells harboring individual constructs were suspended in the infiltration medium to a final concentration of OD_{600} = 0.4 and mixed in a 1:1 or 1:10 ratio prior to leaf infiltration (*PtoDPb1*-NLuc: CLuc-PtoE2Fa, PtoDPb1-NLuc: CLuc-PtoWAK106, *PtoDPb1*-NLuc: CLuc-PtoE2Fa: PtoWAK106-GFP and PtoDPb1-NLuc: CLuc-PtoWAK106: PtoE2Fa-GFP). The plants were placed in the dark for 12 h and then in a growth chamber under normal conditions for a further 24 h. The infiltrated *N. benthamiana* leaves were then sprayed with 100 mM luciferin, incubated in the dark for 10 min, and observed under a low-light cooled charge-coupled device (CCD) imaging apparatus Lumazone_1300B (Roper Bioscience, Sarasota, FL). Each experiment was performed in triplicate.

Method S14 Transcriptional regulation analysis

Electrophoretic mobility shift assay (EMSA)

The EMSA assay was conducted as previously described (Liu *et al.*, 2008). The full-length CDS of *PtoDPb1*$^{\mathrm{Hap1}}$, *PtoDPb1*$^{\mathrm{Hap2}}$, and *PtoE2Fa* were individually inserted into the *pET32a* vector (Zoonbio, Nanjing, China) to produce recombinant *PtoDPb1*$^{\mathrm{Hap1}}$, *PtoDPb1*$^{\mathrm{Hap2}}$, and *PtoE2Fa* in *Escherichia coli* BL21. His-PtoDPb1$^{\mathrm{Hap1,}}$ His-PtoDPb1$^{\mathrm{Hap2}}$, and His-PtoE2Fa fusion proteins were purified using PureCube Ni-NTA Agarose (Cube Biotech, Wayne, PA). DNA motifs representing target gene promoters were synthesized using an EMSA Probe Biotin Labeling Kit (Beyotime, Shanghai, China) by annealing the forward and reverse complementary oligos containing the WTTSSCSS E2F/DP motif. Unlabeled probes were used as competitors. The forward and reverse complementary oligos containing the WTTSSCSS E2F/DP motif are listed in Table S8. A total of 100 ng protein was added in each binding reaction, and when PtoDPb1 and PtoE2Fa proteins were added, each was 600 ng.

Dual-luciferase reporter assay (DLRA)

The CDS of *PtoDPb1*$^{\mathrm{Hap1}}$, *PtoDPb1*$^{\mathrm{Hap2}}$, and *PtoE2Fa* was cloned into the pGreenII 62-SK vector, which was used as an effector plasmid. The *PtoUGT74E2* promoter (2,000 bp upstream) was amplified and cloned into the modified pGreenII 0800-LUC to obtain the reporter plasmids. Each pair of effector and reporter plasmids was transiently co-expressed in *N. benthamiana* leaves, as described by Cheng *et al.* (2017). After 48 h, three leaf discs of 1 cm in diameter were harvested, and the dual-luciferase reporter assay was performed using the Dual-Luciferase Reporter Assay System kit (Promega, Madison, WI) (Niu *et al.*, 2020). The ratio of LUC to REN for both treatments and controls was calculated to test the binding activity of PtoDPb1 to the *PtoUGT74E2* promoter. Each experiment was performed in triplicate.

References

Bradbury PJ, Zhang Z, Kroon DE, Casstevens TM, Ramdoss Y, Buckler ES (2007) TASSEL: software for association mapping of complex traits in diverse samples. *Bioinformatics*, **23**, 2633- 5.

Chen C, Chen H, Zhang Y, Thomas HR, Frank MH, He Y, Xia R (2020) TBtools: An Integrative Toolkit Developed for Interactive Analyses of Big Biological Data. *Mol Plant*, **13**, 1194- 1202.

Cheng MN, Huang ZJ, Hua QZ, Shan W, Kuang JF, Lu WJ, Qin YH, Chen JY (2017) The WRKY transcription factor HpWRKY44 regulates CytP450-like1 expression in red pitaya fruit (*Hylocereus polyrhizus*). *Hortic Res*, **4**, 17039.

Du Q, Lu W, Quan M, Xiao L, Song F, Li P, Zhou D, Xie J, Wang L, Zhang D (2018) GenomeWide Association Studies to Improve Wood Properties: Challenges and Prospects. *Front Plant Sci*, **9**, 1912.

Du Q, Wang B, Wei Z, Zhang D, Li B (2012) Genetic diversity and population structure of Chinese White poplar (*Populus tomentosa*) revealed by SSR markers. *J Hered*, **103**, 853- 862.

Huang Z, Song L, Xiao Y, Zhong X, Wang J, Xu W, Jiang CZ (2022) Overexpression of *Myrothamnus flabellifolia* MfWRKY41 confers drought and salinity tolerance by enhancing root system and antioxidation ability in *Arabidopsis*. *Front Plant Sci*, **13**, 967352.

Langfelder P, Horvath S (2008) WGCNA: An R Package for Weighted Correlation Network Analysis. *BMC Bioinformatics*, **9**, 1- 13.

Langfelder P, Horvath S (2012) Fast R Functions for Robust Correlations and Hierarchical Clustering. *J Stat Softw*, **46**, i11.

Liu H, Yu X, Li K, Klejnot J, Yang H, Lisiero D, Lin C (2008) Photoexcited CRY2 interacts with CIB1 to regulate transcription and floral initiation in *Arabidopsis*. *Science*, **322**, 1535- 1539.

Liu L, Missirian V, Zinkgraf M, Groover A, Filkov V (2014) Evaluation of

experimental design and computational parameter choices affecting analyses of ChIP-seq and RNA-seq data in undomesticated poplar trees. *BMC GENOM*, **15**, S3.

Liu X, Gao Y, Liao J, Miao M, Chen K, Xi F, Wei W, Wang H, Wang Y, Xu X, Reddy ASN, Gu L (2021) Genome-wide profiling of circular RNAs, alternative splicing, and R-loops in stem-differentiating xylem of *Populus trichocarpa*. *J Integr Plant Biol*, **63**, 1294- 1308.

Liu Y, Gu HY, Zhu J, Niu YM, Zhang C, Guo GL (2019) Identification of Hub Genes and Key Pathways Associated With Bipolar Disorder Based on Weighted Gene Co-expression Network Analysis. *Front Physiol*, **10**, 1081.

Lu S, Li Q, Wei H, Chang MJ, Tunlaya-Anukit S, Kim H, Liu J, Song J, Sun YH, Yuan L, Yeh TF, Peszlen I, Ralph J, Sederoff RR, Chiang VL (2013) Ptr-miR397a is a negative regulator of laccase genes affecting lignin content in *Populus trichocarpa*. *Proc Natl Acad Sci USA*, **110**, 10848- 10853.

Lu W, Xiao L, Quan M, Wang Q, El-Kassaby YA, Du Q, Zhang D (2020) Linkage-linkage disequilibrium dissection of the epigenetic quantitative trait loci (epiQTLs) underlying growth and wood properties in *Populus*. *New Phytol*, **225**, 1218- 1233.

Meyer K, Shirley AM, Cusumano JC, Bell-Lelong DA, Chapple C (1998) Lignin monomer composition is determined by the expression of a cytochrome P450-dependent monooxygenase in *Arabidopsis*. *Proc Natl Acad Sci USA*, **95**, 6619-6623.

Niu F, Cui X, Zhao P, Sun M, Yang B, Deyholos MK, Li Y, Zhao X, Jiang YQ (2020) WRKY42 transcription factor positively regulates leaf senescence through modulating SA and ROS synthesis in *Arabidopsis thaliana*. *Plant J*, **104**, 171- 184.

Quan M, Du Q, Xiao L, Lu W, Wang L, Xie J, Song Y, Xu B, Zhang D (2019) Genetic architecture underlying the lignin biosynthesis pathway involves noncoding RNAs and transcription factors for growth and wood properties in *Populus*. *Plant Biotechnol Rep*, **17**, 302- 315.

Quan M, Liu X, Du Q, Xiao L, Lu W, Fang Y, Li P, Ji L, Zhang D (2021) Genome-wide association studies reveal the coordinated regulatory networks

underlying photosynthesis and wood formation in *Populus*. *J Exp Bot*, **72**, 5372-5389.

Sluiter A, Hames B, Ruiz R, Scarlata C, Sluiter J, Templeton D, Crocker DLAP (2008) Determination of structural carbohydrates and lignin in biomass. *LAP*, **1617**, 1-16.

Tamura K, Stecher G, Peterson D, Filipski A, Kumar S (2013) MEGA6: molecular evolutionary genetics analysis version 6.0. *Mol Biol Evol*, **30**, 2725- 2729.

Taylor-Teeples M, Lin L, de Lucas M, Turco G, Toal TW, Gaudinier A, Young NF, Trabucco GM, Veling MT, Lamothe R, Handakumbura PP, Xiong G, Wang C, Corwin J, Tsoukalas A, Zhang L, Ware D, Pauly M, Kliebenstein DJ, Dehesh K, Tagkopoulos I, Breton G, Pruneda-Paz JL, Ahnert SE, Kay SA, Hazen SP, Brady SM (2015) An *Arabidopsis* gene regulatory network for secondary cell wall synthesis. *Nature*, **517**, 571- 575.

Trapnell C, Roberts A, Goff L, Pertea G, Kim D, Kelley DR, Pimentel H, Salzberg SL, Rinn JL, Pachter L (2012) Differential gene and transcript expression analysis of RNA-seq experiments with TopHat and Cufflinks. *Nat Protoc*, **7**, 562-578.

Xiao L, Liu X, Lu W, Chen P, Quan M, Si J, Du Q, Zhang D (2019) Genetic dissection of the gene coexpression network underlying photosynthesis in *Populus*. *Plant Biotechnol-Nar*, **18**, 1015- 1026.

Yavorska, OO, and Burgess S (2017) Mendelianrandomization: an r package for performing mendelian randomization analyses using summarized data. *Int J Epidemiol*, **46**, 1734- 1739.

Zhao DS, Li QF, Zhang CQ, Zhang C, Yang QQ, Pan LX, Ren XY, Lu J, Gu MH, Liu QQ (2018) GS9 acts as a transcriptional activator to regulate rice grain shape and appearance quality. *Nat Commun*, **9**, 1240.

Zhao L, Chen P, Liu P, Song Y, Zhang D (2021) Genetic Effects and Expression Patterns of the Nitrate Transporter (NRT) Gene Family in *Populus tomentosa*. *FRONT Plant Sci*, **12**, 661635.

Zhou H, Lin-Wang K, Liao L, Gu C, Lu Z, Allan AC, Han Y (2015) Peach

MYB7 activates transcription of the proanthocyanidin pathway gene encoding leucoanthocyanidin reductase, but not anthocyanidin reductase. *Front Plant Sci*, **6**, 908.

Zhao L, Chen P, Liu P, Song Y, Zhang D (2021) Genetic Effects and Expression Patterns of the Nitrate Transporter (NRT) Gene Family in *Populus tomentosa*. *FRONT Plant Sci*, **12**, 661635.

Zinkgraf M, Liu L, Groover A, Filkov V (2017) Identifying gene coexpression networks underlying the dynamic regulation of wood-forming tissues in *Populus* under diverse environmental conditions. *New Phytol*, **214**, 1464- 1478.

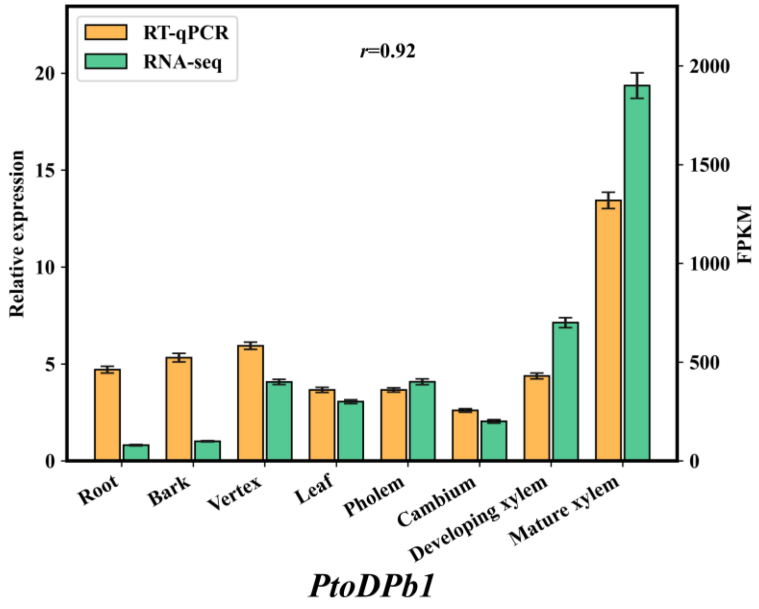

Figure S1. Tissue specific expression analysis of *PtoDPb1*. Pearson correlation coefficient (r) represents correlation analysis of *PtoDPb1* expression levels in different tissues of *P. tomentosa* using RT-qPCR and RNA-seq.

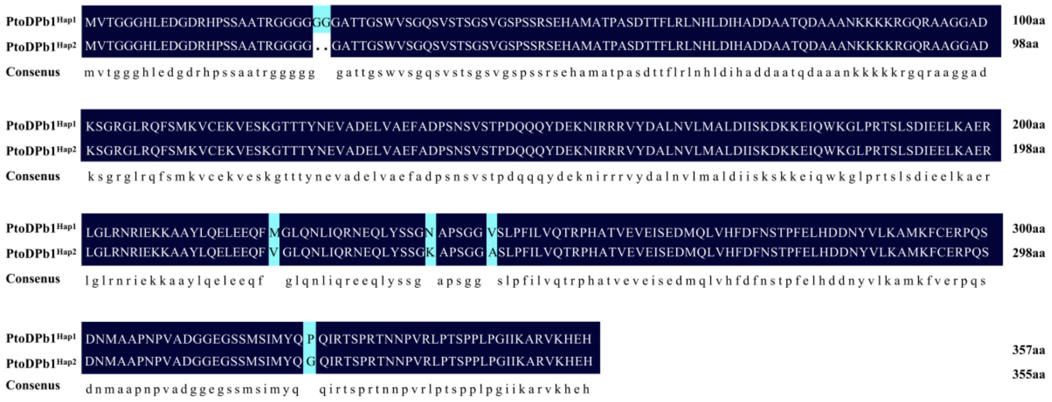

Figure S2. Sequence alignment of PtoDPb1^{Hap1} and PtoDPb1^{Hap2} proteins.

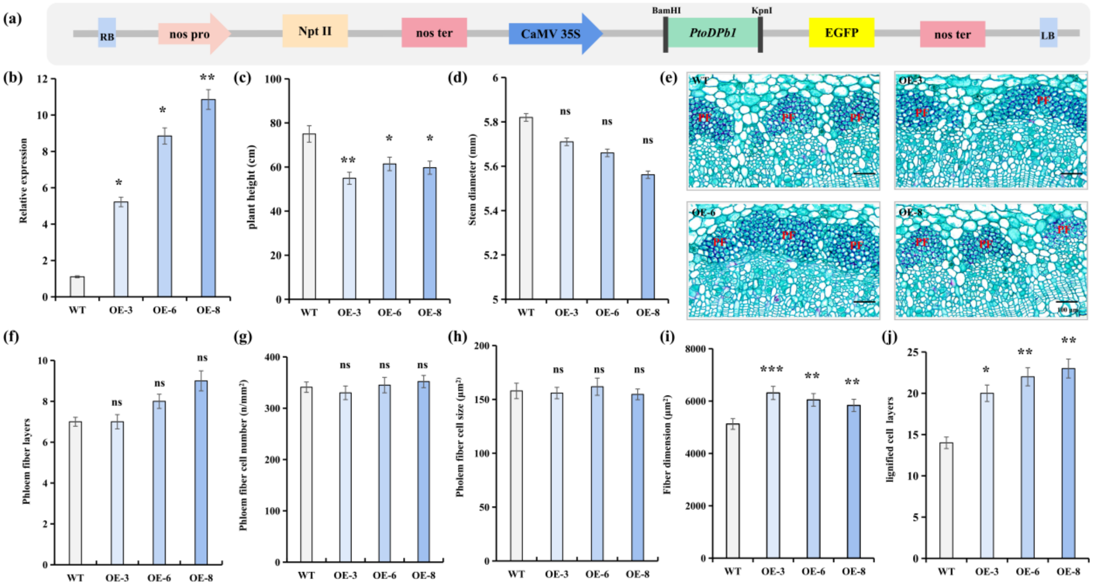

Figure S3. The phenotypes of *PtoDPb1*-overexpressing lines. (a) T-DNA of pBI121-*PtoDPb1* carrying the synthetic *PtoDPb1* gene and NptII gene under the control of the CaMV35S promoter, LB, left border; RB, right border; NptII, neomycin phosphotransferase; nos pro, nopaline synthase promoter; nos term, nopaline synthase terminator. (b) RT-qPCR analysis of *PtoDPb1* transcript level in the wild type (WT) and three independent lines, *35s:PtoDPb1*-3 (OE-3), *35s:PtoDPb1*-6 (OE-6) and *35s:PtoDPb1*-8 (OE-8) plants. (c-d) Plant height (c) and stem diameter (d) of of WT, OE-3, OE-6 and OE-8 plants. (e) Histochemical staining in the stem sections of WT, OE-3, OE-6, and OE-8 plants. Labels PF, refer to phloem fiber cell. (f-h) Quantitative measurement of phloem fiber cell layers (f), number (g) and sizes (h) of WT, OE-3, OE-6, and OE-8 plants. (i) Fiber dimension measurement of WT, OE-3, OE-6, and OE-8 plants. (j) Lignified cell layers' measurement for WT, OE-3, OE-6, and OE-8 plants. Error bars are s.d. and significant differences were determined using *t*-test, * $P < 0.05$, ** $P < 0.01$, *** $P < 0.001$, ns, no significant difference.

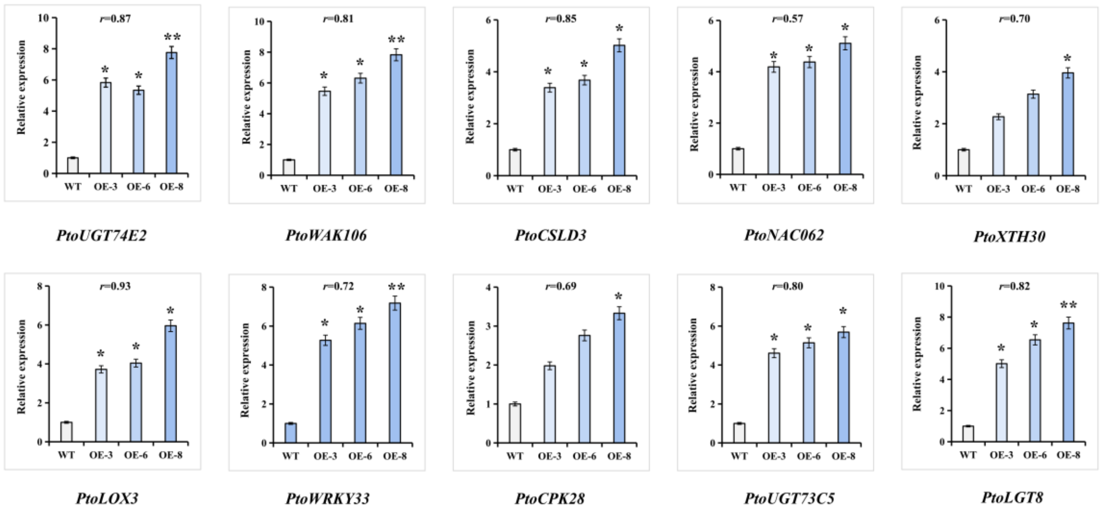

Figure S4. Correlation analysis of expression levels of ten randomly selected differentially expressed genes by the reverse transcription real-time quantitative PCR (RT-qPCR) and RNA-sequencing (RNA-seq). The correlation was showed by Pearson correlation coefficient (*r*).

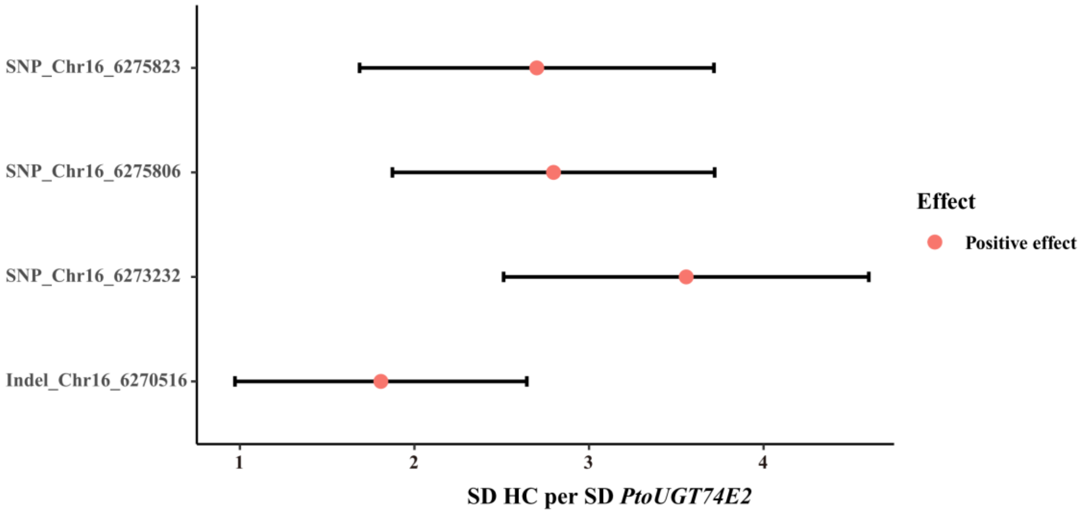

Figure S5. Estimates of the genetic effects of allelic SNPs in *PtoDPb1* on the expression of *PtoUGT74E2* and HC traits. The effect of *PtoUGT74E2* expression on HC was estimated by Mendelian Randomization (MR) using IVW method.

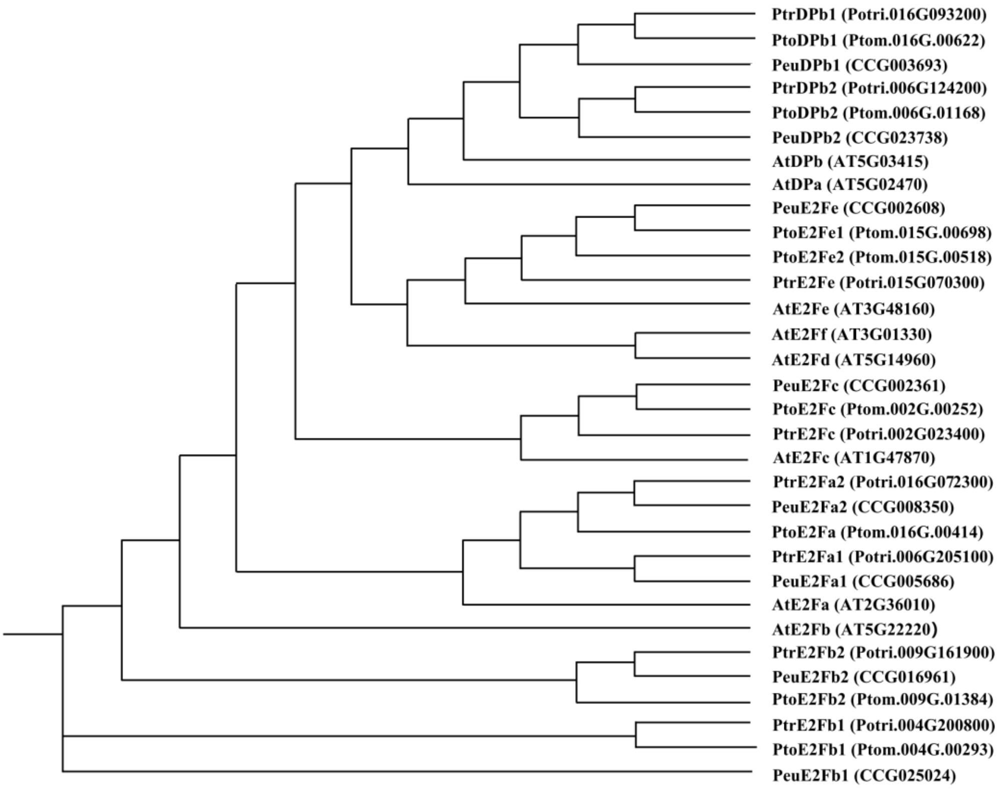

Figure S6. Phylogenetic tree analysis of E2F members and DP members. The phylogenetic tree analysis used *Populus tomentosa*, *Populus trichocarpa*, *Populus euphratica* and *Arabidopsis thaliana*.

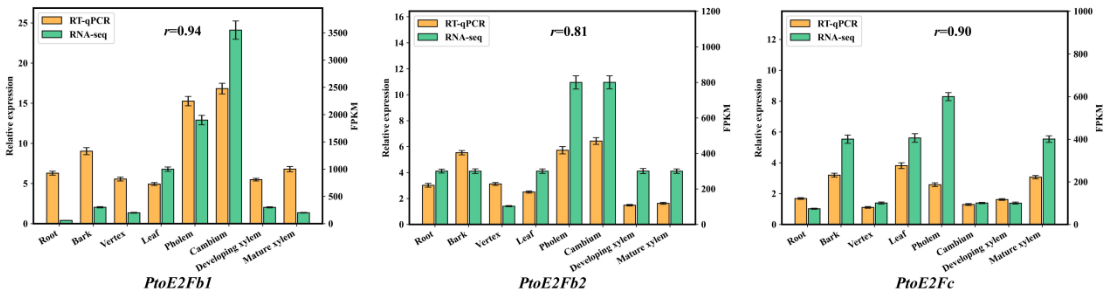

Figure S7. Tissue specific expression analysis of *PtoE2Fb1*, *PtoE2Fb2*, and *PtoE2Fc*. Pearson correlation coefficient (*r*) represents correlation analysis of *PtoE2Fb1*, *PtoE2Fb2*, and *PtoE2Fc* expression levels in different tissues of *P. tomentosa* using the reverse transcription real-time quantitative PCR (RT-qPCR) and RNA-sequencing (RNA-seq), respectively.

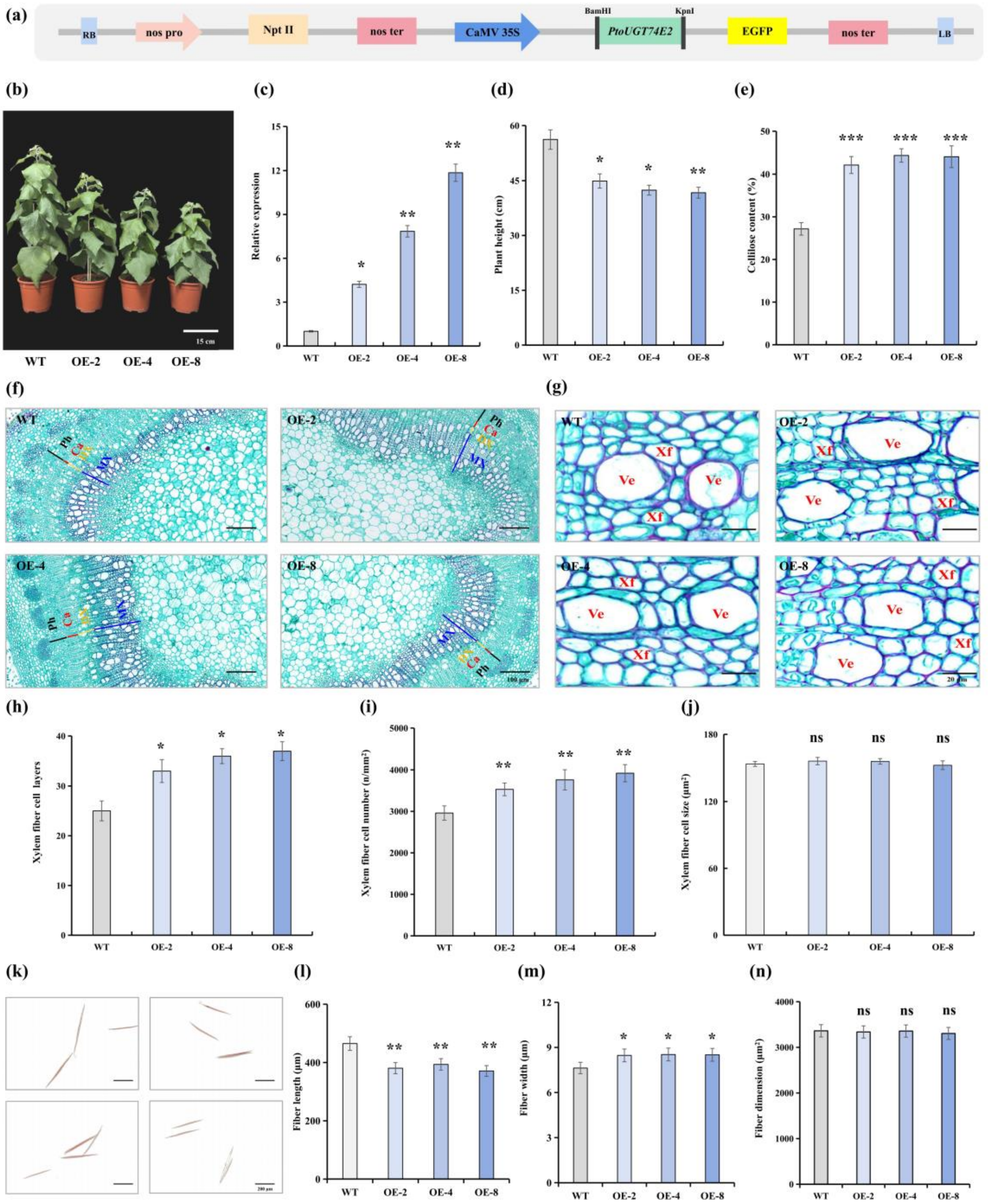

Figure S8. The phenotypes of *PtoUGT74E2*-overexpressing lines. (a) T-DNA of pBI121-*PtoDPb1* carrying the synthetic *PtoUGT74E2* gene and NptII gene under the control of the CaMV35S promoter, LB, left border; RB, right border; NptII, neomycin phosphotransferase; nos pro, nopaline synthase promoter; nos term, nopaline synthase terminator. (b) Phenotypic representations of wild type (WT), *35s:PtoUGT74E2*-2 (OE-2), *35s:PtoUGT74E2*-4 (OE-4), and *35s:PtoUGT74E2*-8 (OE-8) plants. (c) RT-qPCR analysis of *PtoUGT74E2* transcript level in the WT and three independent

lines, OE-2, OE-4 and OE-8 plants. (d-e) Plant height and cellulose content of WT, OE-2, OE-4 and OE-8 plants.(f-g) Histochemical staining in the stem sections of WT, E-2, OE-4 and OE-8 plants. Labels MX, DX, Ph, Ca, Ve, and Xf refer to mature xylem, developing xylem, phloem, cambium, vessel and xylem fiber cell, respectively. (h-j) Quantitative measurement of xylem fiber cell layers (h), number (i) and size (j) of WT, OE-2, OE-4, and OE-8 plants. (k-n) Fiber length (l), fiber width (m) and fiber dimension (n) measurement of WT, OE-2, OE-4, and OE-8 plants. Error bars are s.d. and significant differences were determined using t-test, * $P < 0.05$, ** $P < 0.01$, *** $P < 0.001$, ns, no significant difference.

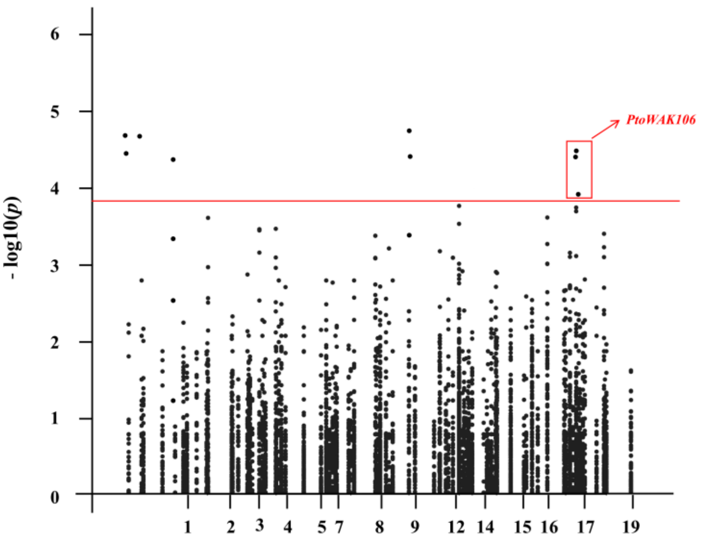

Figure S9. Significantly associated significant single nucleotide polymorphisms (SNPs) of up-stream regulators identified using Expression quantitative trait nucleotide (eQTN) mapping.

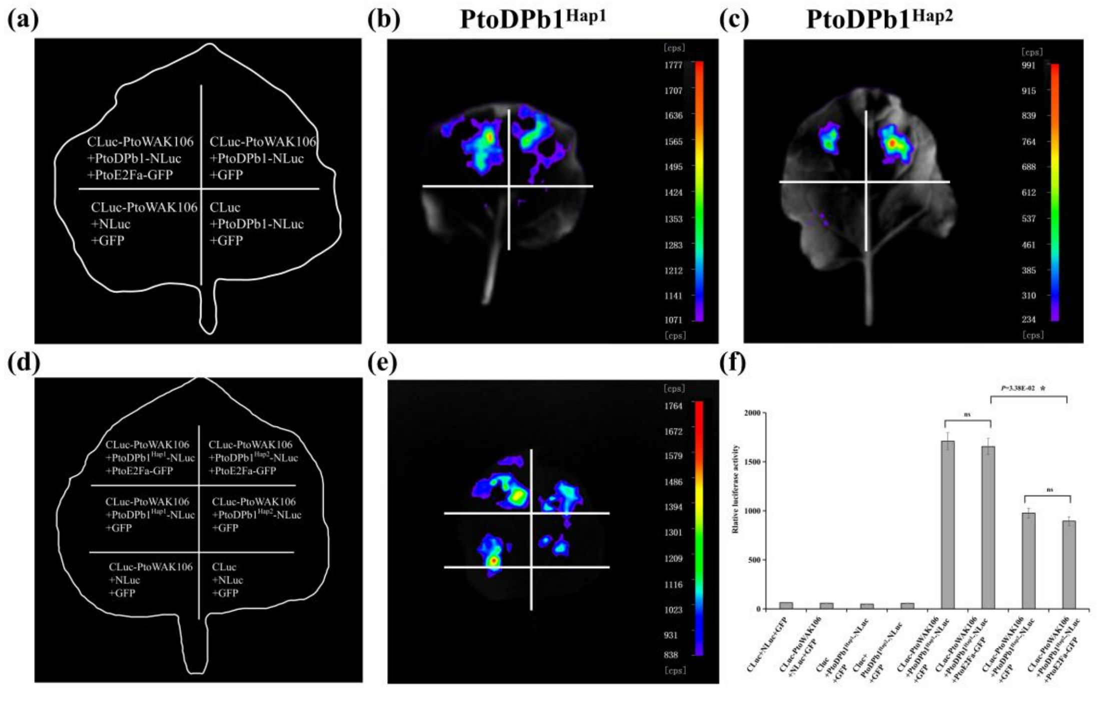

Figure S10. Impact of protein interaction between the PtoE2Fa and PtoWAK106-PtoDPb1. (a-f) Interaction between PtoDPb1$^{Hap1/Hap2}$-NLuc, CLuc-PtoWAK106 and PtoE2Fa-GFP *in vivo* was tested by luciferase bioluminescence imaging (LCI) assay. Firefly luciferase complementation assay in young *N. Benthamiana* leaves. Error bars stand for standard deviation (s.d.) of three biological replicates and significant differences were determined using using *t*-test, * $P < 0.05$, ns, no significant difference.

Table S1. Transcription profiling of RNA-seq datasets used in coexpression analysis.

Experiment	Organism	NCBI SRA and BIG GSA	Number of libraries	Illumina read type
Cambium	*Populus. trichocarpa*	SRP028935	1	50bp SE
Developing xylem	*Populus trichocarpa*	SRP179723	1	50bp SE
Pholem	*Populus trichocarpa*	SRP072680	6	50bp SE
Mature xylem	*Populus trichocarpa*	SRP072680	6	50bp SE
Annual and five-year-old cambium	*Populus tomentosa*	CRA004084	2	50bp SE
Annual and five-year-old pholem	*Populus tomentosa*	CRA004084	2	50bp SE
Annual and five-year-old developing xylem	*Populus tomentosa*	CRA004084	2	50bp SE
Annual and five-year-old mature xylem	*Populus tomentosa*	CRA004084	2	50bp SE

Table S2. Details of significant single nucleotide polymorphisms (SNPs) associated with wood property traits and carbohydrate metabolite traits in the association population of *Populus tomentosa*.

**Traits**	**Associated-SNP**	***P*-value**	**Allele**	**Position**	**Gene annotation**	**Description**
F6P	Chr6_23984270	9.42E-06	G/A	Gene body	Ptom.006G.02713	O-Glycosyl hydrolases family 17 protein, GH17
F6P	Chr6_23984275	9.20E-06	C/T	Gene body	Ptom.006G.02713	O-Glycosyl hydrolases family 17 protein, GH17
F6P	Chr6_23984277	9.12E-06	C/G	Gene body	Ptom.006G.02713	O-Glycosyl hydrolases family 17 protein, GH17
F6P	Chr6_23984282	5.09E-05	G/A	Gene body	Ptom.006G.02713	O-Glycosyl hydrolases family 17 protein, GH17
FL	Chr6_4919352	2.10E-06	T/C	Gene body	Ptom.006G.00618	Glycosyl hydrolase superfamily protein
LF	Chr6_23984270	2.26E-06	G/A	Gene body	Ptom.006G.02713	O-Glycosyl hydrolases family 17 protein, GH17
LF	Chr6_23984275	2.60E-05	C/T	Gene body	Ptom.006G.02713	O-Glycosyl hydrolases family 17 protein, GH17
LF	Chr6_23984277	2.20E-05	C/G	Gene body	Ptom.006G.02713	O-Glycosyl hydrolases family 17 protein, GH17
LF	Chr6_23984282	1.13E-05	G/A	Gene body	Ptom.006G.02713	O-Glycosyl hydrolases family 17 protein, GH17
T6P	Chr6_23984270	1.34E-05	G/A	Gene body	Ptom.006G.02713	O-Glycosyl hydrolases family 17 protein, GH17
T6P	Chr6_23984275	1.40E-05	C/T	Gene body	Ptom.006G.02713	O-Glycosyl hydrolases family 17 protein, GH17
T6P	Chr6_23984277	1.34E-04	C/G	Gene body	Ptom.006G.02713	O-Glycosyl hydrolases family 17 protein, GH17
T6P	Chr6_23984282	5.97E-06	G/A	Gene body	Ptom.006G.02713	O-Glycosyl hydrolases family 17 protein, GH17
HEC	Chr6_42862727	6.61E-06	C/T	Promoter	Ptom.001G.03755	Early nodulin-like protein 1, ENODL1
G6P	Chr6_41430317	2.63E-07	G/A	Promoter	Ptom.001G.03653	Uridine diphosphate glycosyltransferase 74E2, UGT74E2
FL	Chr5_4807736	1.70E-05	G/C	Gene body	Ptom.005G.00629	White-brown complex homolog protein 11, WBC11
LF	Chr5_19179425	9.63E-06	T/C	Flanking region	Ptom.005G.02045	Actin depolymerizing factor 6, ADF6
T6P	Chr5_19179425	7.78E-06	T/C	Flanking region	Ptom.005G.02045	Actin depolymerizing factor 6, ADF6
FL	Chr4_6758715	2.10E-06	A/G	Flanking region	Ptom.004G.00824	Cytochrome P450, family 707, subfamily A, polypeptide 4, CYP707A4
F6P	Chr11_5435991	8.19E-06	C/T	Promoter	Ptom.011G.00378	NTF2-like, NTL
FL	Chr11_11442596	2.10E-05	T/C	Gene body	Ptom.011G.00703	Glucan synthase-like 4, GSL04

HEC	Chr11_15637581	5.24E-05	G/A	Gene body	Ptom.011G.01096	Actin 7, ACT7
CC	Chr11_13436532	1.67E-05	A/T	Gene body	Ptom.011G.00861	Seven transmembrane MLO family protein, MLO11
MFA	Chr16_4062968	1.12E-05	A/T	Promoter	Ptom.016G.00407	Double Clp-N motif-containing P-loop nucleoside triphosphate hydrolases superfamily protein, SMXL3
G6P	Chr16_6268473	9.37E-06	T/C	Promoter	Ptom.016G.00622	E2F/DP transcription factor family member, DPb1
HC	Chr16_6273232	1.33E-05	A/G	Gene body	Ptom.016G.00622	E2F/DP transcription factor family member, DPb1
HC	Chr16_6275823	6.05E-06	T/C	Gene body	Ptom.016G.00622	E2F/DP transcription factor family member, DPb1
HC	Chr16_6275806	4.14E-06	T/G	Gene body	Ptom.016G.00622	E2F/DP transcription factor family member, DPb1
T6P	Chr16_6269303	8.08E-06	C/T	Promoter	Ptom.016G.00622	E2F/DP transcription factor family member, DPb1
FL	Chr19_12801084	2.10E-06	A/T	Gene body	Ptom.019G.00719	Homeodomain-like superfamily protein, MYR2
HEC	Chr13_9690166	2.84E-05	G/A	Flanking region	Ptom.013G.00771	Glutamate dehydrogenase 1, GDH1
HEC	Chr14_6160329	5.81E-06	T/G	Gene body	Ptom.014G.00751	ADP-ribosylation factor A1E, ARFA1E
HEC	Chr14_6160338	3.16E-05	G/A	Gene body	Ptom.014G.00751	ADP-ribosylation factor A1E, ARFA1E
LC	Chr14_6160391	6.69E-06	A/G	Gene body	Ptom.014G.00751	ADP-ribosylation factor A1E, ARFA1E
HC	Chr14_6160338	1.78E-05	G/A	Gene body	Ptom.014G.00751	ADP-ribosylation factor A1E, ARFA1E

Table S3. The 68 connected genes with *PtoDPb1* using weighted gene co-expression network analysis (WGCNA).

**From Node**	**To Node**	**Orthologous genes of *Populus trichocarpa***	**Weight**	**Description**
Ptom.016G.00622	Ptom.001G.02974	Potri.001G309700	0.21	RING/U-box superfamily protein, PRU2
Ptom.016G.00622	Ptom.004G.00824	Potri.004G140900	0.20	Cytochrome P450, family 707, subfamily A, polypeptide 4, CYP707A4
Ptom.016G.00622	Ptom.004G.01377	Potri.004G075200	0.21	Glutamate decarboxylase, GAD1
Ptom.016G.00622	Ptom.007G.00322	Potri.007G031700	0.22	Galacturonosyltransferase-like 2, GATL2
Ptom.016G.00622	Ptom.009G.00386	Potri.009G053900	0.22	MYB domain protein 46, MYB46
Ptom.016G.00622	Ptom.003G.01202	Potri.003G096100	0.22	Transcription factor jumonji family protein /zinc finger (C5HC2 type) family protein, JMJ13
Ptom.016G.00622	Ptom.008G.01502	Potri.008G187900	0.22	Alpha/beta-Hydrolases superfamily protein, LDAH1
Ptom.016G.00622	Ptom.016G.00361	Potri.016G066700	0.20	Calmodulin-domain protein kinase 2, CPK2
Ptom.016G.00622	Ptom.019G.00093	Potri.019G128100	0.27	SOS2-like protein kinase of the CBL-interacting protein, CIPK11
Ptom.016G.00622	Ptom.005G.01032	Potri.005G113600	0.26	Calcium-dependent protein kinase 28, CPK28
Ptom.016G.00622	Ptom.014G.00124	Potri.014G022500	0.26	MYB domain protein 73, MYB73
Ptom.016G.00622	Ptom.002G.01718	Potri.002G154000	0.22	NAC domain transcriptional regulator superfamily protein 14, NAC014
Ptom.016G.00622	Ptom.001G.01316	Potri.001G136100	0.25	Xyloglucan endotransglucosylase/hydrolase 30, XTH30
Ptom.016G.00622	Ptom.015G.00836	Potri.015G082700	0.21	MYB domain protein 50, MYB50
Ptom.016G.00622	Ptom.004G.02000	Potri.011G057000	0.21	Ethylene responsive element binding factor 9, ERF9
Ptom.016G.00622	Ptom.001G.01317	Potri.001G136200	0.22	Cellulose synthase-like D3, CSLD3
Ptom.016G.00622	Ptom.009G.00474	Potri.009G063900	0.22	Positive stress responses regulator, SAP5
Ptom.016G.00622	Ptom.007G.00793	Potri.007G099400	0.30	NAC domain transcriptional regulator superfamily protein, NAC002

Ptom.016G.00622	Ptom.001G.03811	Potri.001G406600	0.21	Cotton fiber-like protein, DUF761
Ptom.016G.00622	Ptom.001G.00808	Potri.001G083500	0.20	Basic helix-loop-helix (bHLH) DNA-binding family protein, MYC2
Ptom.016G.00622	Ptom.001G.03728	Potri.001G397200	0.22	Ethylene responsive element binding factor 4, ERF4
Ptom.016G.00622	Ptom.001G.01689	Potri.001G167700	0.22	Lipoxygenase 3, LOX3
Ptom.016G.00622	Ptom.001G.02128	Potri.001G219100	0.22	Duplicated homeodomain-like superfamily protein, DIV2
Ptom.016G.00622	Ptom.009G.00561	Potri.009G073000	0.22	Protein phosphatase 2C family protein, P2C1
Ptom.016G.00622	Ptom.001G.03653	Potri.001G389200	0.20	Uridine diphosphate glycosyltransferase 74E2, UGT74E2
Ptom.016G.00622	Ptom.002G.00062	Potri.002G004900	0.21	Protein kinase superfamily protein, PKS1
Ptom.016G.00622	Ptom.005G.02597	Potri.005G259900	0.26	Calmodulin-like 38, CML38
Ptom.016G.00622	Ptom.001G.01477	Potri.001G154200	0.26	Ethylene responsive element binding factor 5, ERF5
Ptom.016G.00622	Ptom.005G.02594	Potri.005G259600	0.20	Protein kinase superfamily protein, LYK5
Ptom.016G.00622	Ptom.001G.03803	Potri.001G404100	0.22	NAC domain transcriptional regulator superfamily protein 72, NAC072
Ptom.016G.00622	Ptom.008G.01547	Potri.008G192600	0.22	Glucosyl transferase family 8, LGT8
Ptom.016G.00622	Ptom.008G.00538	Potri.008G055900	0.21	O-Glycosyl hydrolases family 17 protein
Ptom.016G.00622	Ptom.012G.00519	Potri.012G007500	0.22	NAC domain transcriptional regulator superfamily protein 62, NAC062
Ptom.016G.00622	Ptom.012G.00154	Potri.012G048700	0.22	UDP-glucosy transferase 73C5, UGT73C5
Ptom.016G.00622	Ptom.015G.00878	Potri.015G086800	0.20	Leucine-rich repeat protein kinase family protein, EVR
Ptom.016G.00622	Ptom.005G.00239	Potri.005G032700	0.21	Lipoxygenase 1, LOX1
Ptom.016G.00622	Ptom.017G.00439	Potri.015G121900	0.26	Protein kinase superfamily protein, WAK106
Ptom.016G.00622	Ptom.001G.03410	Potri.001G472800	0.22	WRKY DNA-binding protein 2, WRKY2
Ptom.016G.00622	Ptom.002G.01367	Potri.002G127000	0.25	Calmodulin like 37, CML37
Ptom.016G.00622	Ptom.016G.01025	Potri.004G175000	0.30	Ubiquitin-conjugating enzyme 10, UBC10
Ptom.016G.00622	Ptom.009G.00056	Potri.009G095400	0.33	UDP-Glycosyltransferase superfamily protein, UGT84A1

Ptom.016G.00622	Ptom.010G.02141	Potri.010G039800	0.34	CLAVATA3/ESR-RELATED 26, CLE26
Ptom.016G.00622	Ptom.001G.04207	Potri.011G140700	0.33	Glutathione S-transferase TAU 19, GST8
Ptom.016G.00622	Ptom.006G.01215	Potri.014G087400	0.30	Syntaxin of plants 71, SYP71
Ptom.016G.00622	Ptom.017G.00214	Potri.017G131100	0.30	Glutamine synthase clone R1, GLN1
Ptom.016G.00622	Ptom.018G.00496	Potri.018G006032	0.21	H(+)-ATPase 5, HA5
Ptom.016G.00622	Ptom.001G.00073	Potri.001G006000	0.26	HVA22-like protein F, HVA22F
Ptom.016G.00622	Ptom.001G.01705	Potri.001G169600	0.26	MYB domain protein 23, MYB23
Ptom.016G.00622	Ptom.002G.00180	Potri.002G017000	0.22	Calcium-dependent protein kinase 33, CPK33
Ptom.016G.00622	Ptom.002G.00636	Potri.002G058700	0.26	Glucan synthase-like 7, GSL07
Ptom.016G.00622	Ptom.003G.00747	Potri.003G142400	0.22	Cellulose synthase like G3, CSLG3
Ptom.016G.00622	Ptom.003G.00216	Potri.003G198400	0.22	Multidrug resistance-associated protein 3, MRP3
Ptom.016G.00622	Ptom.004G.00943	Potri.004G139900	0.26	phosphatidylinositol-4-phosphate 5-kinase 1, PIP5K1
Ptom.016G.00622	Ptom.005G.02249	Potri.005G245000	0.20	Calcium-dependent protein kinase 29, CPK29
Ptom.016G.00622	Ptom.006G.02687	Potri.006G277800	0.23	Basic leucine zipper 9, BZIP9
Ptom.016G.00622	Ptom.008G.01516	Potri.008G189400	0.21	Galactinol synthase 4, Gols4
Ptom.016G.00622	Ptom.008G.01564	Potri.008G194100	0.26	Thioredoxin H-type 8, TH8
Ptom.016G.00622	Ptom.009G.01378	Potri.009G161300	0.10	NTM1-like 8, NTL8
Ptom.016G.00622	Ptom.011G.00703	Potri.011G095100	0.23	Glucan synthase-like 4, GSL04
Ptom.016G.00622	Ptom.012G.01114	Potri.012G120400	0.21	Phloem protein 2-A1, PP2-A1
Ptom.016G.00622	Ptom.013G.00316	Potri.013G115200	0.20	Sucrose-proton symporter 2, SUC2
Ptom.016G.00622	Ptom.014G.01617	Potri.014G177200	0.24	PLAT/LH2 domain-containing lipoxygenase family protein, LOX5
Ptom.016G.00622	Ptom.015G.01069	Potri.015G120200	0.30	Phloem protein 2-A10, PP2-A10
Ptom.016G.00622	Ptom.017G.00498	Potri.017G017100	0.23	C2H2 and C2HC zinc fingers superfamily protein, MGP
Ptom.016G.00622	Ptom.017G.00960	Potri.017G074600	0.29	Clavata3/esr-related 25, CL325
Ptom.016G.00622	Ptom.017G.00295	Potri.017G139100	0.29	Sucrose synthase 5, SUS5

Ptom.016G.00622	Ptom.019G.00802	Potri.019G040900	0.29	MYB domain protein 105, MYB105
Ptom.016G.00622	Ptom.019G.00346	Potri.019G076800	0.22	The MADS box family of transcription factors, XAL1

Table S4. The 36 Overlapping genes detected using weighted gene co-expression network analysis (WGCNA) and RNA-sequencing (RNA-seq) analysis.

**Genes of *Populus tomentosa***	**Orthologous genes of *Populus trichocarpa***	**Weight**	**Log_2 Fold Change**	***P*_value**	***q*_value**	**Description**
Ptom.008G.01564	Potri.008G194100	0.26	-3.2574	5.00E-05	5.70E-03	Thioredoxin H-type 8, TH8
Ptom.001G.02974	Potri.001G309700	0.21	-2.31324	2.00E-04	1.81E-02	RING/U-box superfamily protein, PRU2
Ptom.004G.00824	Potri.004G140900	0.20	-1.84103	3.00E-04	2.51E-02	Cytochrome P450, family 707, subfamily A, polypeptide 4, CYP707A4
Ptom.004G.01377	Potri.004G075200	0.21	-1.7067	4.50E-04	3.44E-02	Glutamate decarboxylase, GAD1
Ptom.007G.00322	Potri.007G031700	0.22	1.47526	6.50E-04	4.39E-02	Galacturonosyltransferase-like 2, GATL2
Ptom.009G.00386	Potri.009G053900	0.22	1.54308	4.00E-04	3.14E-02	MYB domain protein 46, MYB46
Ptom.003G.01202	Potri.003G096100	0.22	2.01014	5.00E-05	2.32E-04	Transcription factor jumonji family protein /zinc finger (C5HC2 type) family protein, JMJ13
Ptom.008G.01502	Potri.008G187900	0.22	1.78943	7.50E-04	4.85E-02	Alpha/beta-Hydrolases superfamily protein, LDAH1
Ptom.016G.00361	Potri.016G066700	0.20	1.97688	5.50E-04	3.96E-02	Calmodulin-domain protein kinase 2, CPK2
Ptom.019G.00093	Potri.019G128100	0.27	2.09033	1.00E-04	1.14E-02	SOS2-like protein kinase of the CBL-interacting protein, CIPK11
Ptom.005G.01032	Potri.005G113600	0.26	2.12818	2.50E-04	2.19E-02	Calcium-dependent protein kinase 28, CPK28
Ptom.014G.00124	Potri.014G022500	0.26	2.09512	5.00E-05	6.88E-03	MYB domain protein 73, MYB73
Ptom.002G.01718	Potri.002G154000	0.22	2.22102	5.00E-04	3.73E-02	NAC domain transcriptional regulator superfamily protein 14, NAC014
Ptom.001G.01316	Potri.001G136100	0.25	2.27812	2.00E-04	1.81E-02	Xyloglucan endotransglucosylase/hydrolase 30, XTH30
Ptom.015G.00836	Potri.015G082700	0.21	2.33455	5.00E-05	6.88E-03	MYB domain protein 50, MYB50
Ptom.004G.02000	Potri.011G057000	0.21	4.36372	5.50E-04	3.96E-02	Ethylene responsive element binding factor 9, ERF9
Ptom.001G.01317	Potri.001G136200	0.22	2.73708	5.00E-05	6.88E-03	Cellulose synthase-like D3, CSLD3
Ptom.009G.00474	Potri.009G063900	0.22	2.5968	5.00E-05	6.88E-03	Positive stress responses regulator, SAP5

Ptom.007G.00793	Potri.007G099400	0.30	2.41524	6.00E-04	4.17E-02	NAC domain transcriptional regulator superfamily protein, NAC002
Ptom.001G.03811	Potri.001G406600	0.21	2.67442	3.50E-04	2.84E-02	Cotton fiber-like protein, DUF761
Ptom.001G.00808	Potri.001G083500	0.20	2.77559	6.00E-04	4.14E-02	Basic helix-loop-helix (bHLH) DNA-binding family protein, MYC2
Ptom.001G.03728	Potri.001G397200	0.22	2.90476	5.00E-05	6.88E-03	Ethylene responsive element binding factor 4, ERF4
Ptom.001G.01689	Potri.001G167700	0.22	2.95014	5.00E-05	6.88E-03	Lipoxygenase 3, LOX3
Ptom.001G.02128	Potri.001G219100	0.22	3.0599	5.00E-05	5.70E-03	Duplicated homeodomain-like superfamily protein, DIV2
Ptom.009G.00561	Potri.009G073000	0.22	3.15528	1.00E-04	1.06E-02	Protein phosphatase 2C family protein, P2C1
Ptom.001G.03653	Potri.001G389200	0.20	3.30466	5.00E-05	5.70E-03	Uridine diphosphate glycosyltransferase 74E2, UGT74E2
Ptom.001G.01477	Potri.001G154200	0.26	6.02183	5.00E-05	6.88E-03	Ethylene responsive element binding factor 5, ERF5
Ptom.005G.02594	Potri.005G259600	0.20	3.74662	5.00E-05	6.88E-03	Protein kinase superfamily protein, LYK5
Ptom.001G.03803	Potri.001G404100	0.22	4.11645	5.00E-05	6.88E-03	NAC domain transcriptional regulator superfamily protein 72, NAC072
Ptom.008G.01547	Potri.008G192600	0.22	4.51198	5.00E-05	6.88E-03	Glucosyl transferase family 8, LGT8
Ptom.008G.00538	Potri.008G055900	0.21	4.61847	5.00E-05	6.88E-03	O-Glycosyl hydrolases family 17 protein
Ptom.012G.00519	Potri.012G007500	0.22	4.46713	5.00E-05	6.88E-03	NAC domain transcriptional regulator superfamily protein 62, NAC062
Ptom.012G.00154	Potri.012G048700	0.22	4.61652	4.00E-04	3.16E-02	UDP-glucosy transferase 73C5, UGT73C5
Ptom.015G.00878	Potri.015G086800	0.20	5.75947	5.00E-05	6.88E-03	Leucine-rich repeat protein kinase family protein, EVR
Ptom.005G.00239	Potri.005G032700	0.21	3.36849	5.00E-05	5.70E-03	Lipoxygenase 1, LOX1
Ptom.017G.00439	Potri.015G121900	0.26	6.20	5.00E-05	4.57E-03	Protein kinase superfamily protein, WAK106

Table S5. Down-stream genes identified using expression quantitative trait nucleotide (eQTN) mapping.

**Traits（Expression levels of genes）**	**Associated SNP/Indel**	***P*-value**	***Q*-value**	**Allele**	**Position**	**Gene annotation**	***r*[a]**	***r*[b]**	**Description**
PtoUGT74E2	Chr16_6268865	7.77E-05	1.75E-04	G/A	Promoter	Ptom.001G.03653	0.916	0.901	Uridine diphosphate glycosyltransferase 74E2, UGT74E2
PtoUGT74E2	Chr16_6273232	1.78E-04	3.71E-03	A/G	Gene body	Ptom.001G.03653	0.916	0.901	Uridine diphosphate glycosyltransferase 74E2, UGT74E2
PtoUGT74E2	Chr16_6269413	2.77E-04	7.77E-03	T/C	Promoter	Ptom.001G.03653	0.916	0.901	Uridine diphosphate glycosyltransferase 74E2, UGT74E2
PtoUGT74E2	Chr16_6269368	1.13E-04	9.09E-03	C/T	Promoter	Ptom.001G.03653	0.916	0.901	Uridine diphosphate glycosyltransferase 74E2, UGT74E2
PtoUGT74E2	Chr16_6270591	1.17E-04	1.14E-03	A/T	Gene body	Ptom.001G.03653	0.916	0.901	Uridine diphosphate glycosyltransferase 74E2, UGT74E2
Ptom.008G.00538	Chr16_6269257	1.20E-04	1.50E-03	T/C	Promoter	Ptom.008G.00538	0.855	0.823	O-Glycosyl hydrolases family 17 protein
PtoXTH30	Chr16_6276705	1.33E-04	1.04E-03	C/T	Flanking region	Ptom.001G.01316	-0.835	-0.808	Xyloglucan endotransglucosylase/hydrolase30, XTH30
PtoUGT74E2	Chr16_6275806	1.49E-04	2.83E-03	T/G	Gene body	Ptom.001G.03653	0.916	0.901	Uridine diphosphate glycosyltransferase 74E2, UGT74E2

PtoUGT74E2	Chr16_6269425	1.85E-04	3.48E-03	G/A	Promoter	Ptom.001G.03653	0.916	0.901	Uridine diphosphate glycosyltransferase 74E2, UGT74E2
PtoLGT8	Chr16_6269257	7.33E-04	8.59E-03	T/C	Promoter	Ptom.008G.01547	0.866	0.824	Glucosyl transferase family8, LGT8
PtoUGT74E2	Chr16_6270516	3.29E-05	4.37E-04	G/-	Gene body	Ptom.001G.03653	0.916	0.901	Uridine diphosphate glycosyltransferase 74E2, UGT74E2
PtoLGT8	Chr16_6269465	1.69E-04	5.59E-03	T/-	Promoter	Ptom.008G.01547	0.866	0.824	Glucosyl transferase family8, LGT8

[a]Pearson correlation coefficients in vascular tissues of *P. tomentosa*

[b]Pearson correlation coefficients in mature xylem of six *P. trichocarpa* individuals

Table S6. Mendelian Randomization (MR) results of the relationship of allelic SNPs of *PtoDPb1*, expression of *PtoUGT74E2* and HC traits.

**Gene ID**	**SNP/Indel**	**exposure**	**outcome**	**Estimate**	**Std**	**Error**	**95% Ci**	***P*-value**
PtoDPb1	Chr16_6273232	expression of *PtoUGT74E2*	HC	3.52	1.046	1.47	5.57	0.001
PtoDPb1	Chr16_6275806	expression of *PtoUGT74E2*	HC	2.76	0.923	0.951	4.569	0.003
PtoDPb1	Chr16_6275823	expression of *PtoUGT74E2*	HC	2.664	1.015	0.674	4.654	0.009
PtoDPb1	Chr16_6270516	expression of *PtoUGT74E2*	HC	1.771	0.836	0.133	3.409	0.034

Table S7. Up-stream regulators identified using expression quantitative trait nucleotide (eQTN) mapping.

**Traits （Expression levels of genes）**	**Associated SNP**	***P*-value**	**allele**	**Position**	**Gene annotation**	***r*[a]**	***r*[b]**	**Description**
PtoDPb1	Chr1_15720995	3.10E-05	T/C	Gene body	Ptom.001G.01689	0.905	0.893	Lipoxygenase3, LOX3
PtoDPb1	Chr1_15721039	5.10E-05	A/G	Gene body	Ptom.001G.01689	0.905	0.893	Lipoxygenase3, LOX3
PtoDPb1	Chr1_23563343	3.09E-05	A/G	Promoter	Ptom.001G.02128	0.406	0.541	Duplicated homeodomain-like superfamily protein, DIV2
PtoDPb1	Chr1_43665822	6.38E-05	T/C	Gene body	Ptom.001G.03811	0.849	0.886	Cotton fiber-like protein, DUF761
PtoDPb1	Chr17_4364064	6.46E-05	C/T	Promoter	Ptom.017G.00439	0.928	0.903	Wall associated kinase106, WAK106
PtoDPb1	Chr17_4365013	1.37E-04	C/G	Gene body	Ptom.017G.00439	0.928	0.903	Wall associated kinase106, WAK106
PtoDPb1	Chr17_4365989	5.74E-05	A/C	Gene body	Ptom.017G.00439	0.928	0.903	Wall associated kinase106, WAK106
PtoDPb1	Chr9_4208098	2.13E-05	G/A	Gene body	Ptom.009G.00474	0.868	0.906	Positive stress responses regulator, SAP5
PtoDPb1	Chr9_4208120	3.08E-05	C/T	Gene body	Ptom.009G.00474	0.868	0.906	Positive stress responses regulator, SAP5

[a]Pearson correlation coefficients in vascular tissues of *P. tomentosa*

[b]Pearson correlation coefficients in mature xylem of six *P. trichocarpa* individuals

Table S8. The oligonucleotide sequences of primers used in this study.

Primer Name	Sequence (5'-3')	Description
PtoDPb1-F	ATGGTTACTGGCGGGGGC	CDS for PtoDPb1
PtoDPb1-R	ATGCTCATGCTTAACACGTGCTTTT	CDS for PtoDPb1
PtoDPb1$^{\triangle HD}$-F	ATGGTTACTGGCGGGGGC	CDS for PtoDPb1$^{\triangle HD}$
PtoDPb1$^{\triangle HD}$-R	ACCTTTCCACTGTATTTCCTTTTTA	CDS for PtoDPb1$^{\triangle HD}$
PtoE2Fa-F	ATGTCCGGCGGCGCTAAA	CDS for PtoE2Fa2
PtoE2Fa-R	TAGAGTTTGTGTCCTAGGAGTTG	CDS for PtoE2Fa2
PtoWAK106-F	ATGTCCGTTGACGAGCTTGACT	CDS for PtoWAK106
PtoWAK106-R	ATAAATACCTTTTGGCATAATAGAA	CDS for PtoWAK106
18s rRNA-F	GGCATGGAAGGTGATGCAGATC	RT-qPCR
18s rRNA-R	CTGTGTCAAACAAGAACTTGTCC	RT-qPCR
PtoDPb1-qpc-F	ACCATGACAACACCAACCAGGATG	RT-qPCR
PtoDPb1-qpc-R	AGCGTGAAACAGGGGAAGAAGATG	RT-qPCR
PtoUGT74E2-qpc-F	TACCAGGCAACTTCGTAGAGGAGAC	RT-qPCR
PtoUGT74E2-qpc-R	CGGATTTGTGAGCCAGGACCTTTAG	RT-qPCR
PtoWAK106-qpc-F	ACCTCTTTGGTGGTGCGTATGC	RT-qPCR
PtoWAK106-qpc-R	TGGATATTGCCGCAAGAAGAAGGAG	RT-qPCR
PtoCSLD3-qpc-F	TGACCGAGTTCCAAACAAGCCATC	RT-qPCR
PtoCSLD3-qpc-R	CCTGAGCATCACAACCCGACATC	RT-qPCR
PtoNAC062-qpc-F	AACTGCTGATTTACCCGCTTCTGAG	RT-qPCR
PtoNAC062-qpc-R	CTGCTTCTTGGACCTGCTCATGG	RT-qPCR
PtoXTH30-qpc-F	TGCCACCACCAGAATGTGTTGTG	RT-qPCR
PtoXTH30-qpc-R	GCTGCCACCGAATCTCAATCTACC	RT-qPCR
PtoLOX3-qpc-F	GCACCTATGTTGAGCGTTACTACCC	RT-qPCR

PtoLOX3-qpc-R	CCACCAGTCAGCATCACGAAGATC	RT-qPCR
PtoWRKY33-qpc-F	GGTGGTGATGACTTGGATGAGGATG	RT-qPCR
PtoWRKY33-qpc-R	TCTGCTGCCAGGTGCTGAAATTC	RT-qPCR
PtoCPK28-qpc-F	TGGGTCAGAGAAGGAGGAGTTGC	RT-qPCR
PtoCPK28-qpc-R	TTCATCAATCGTGCTTGCCAATGC	RT-qPCR
PtoUGT73C5-qpc-F	AGGAGGTAAAGTTTGGTGCGTTGG	RT-qPCR
PtoUGT73C5-qpc-R	CAAGCCACTTCAAGCATTCCGATTC	RT-qPCR
PtoLGT8-qpc-F	TGATGGACTTGGTGAGGTGGAGAG	RT-qPCR
PtoLGT8-qpc-R	GGCAGTGAACCCAACTCGTATATCC	RT-qPCR
PtoE2Fa-qpc-F	ACATTGCAGCCGAAACCTTGGAG	RT-qPCR
PtoE2Fa-qpc-R	CTACCTGCCTTGGCCTTGAAGC	RT-qPCR
PtoE2Fb1-qpc-F	ATGTGGCGTAACGAACCTGTGG	RT-qPCR
PtoE2Fb1-qpc-R	GATGGTGGAGTTTGGGGCTGTG	RT-qPCR
PtoE2Fb2-qpc-F	ACCACCTCCAAGCGACTCGTC	RT-qPCR
PtoE2Fb2-qpc-R	TTGCTCTGCTCTCCTCTGTGACC	RT-qPCR
PtoE2Fc-qpc-F	CTCACCACCTGCCTCCTCCTC	RT-qPCR
PtoE2Fc-qpc-R	TTATTCCCAGCGCAACGTCACG	RT-qPCR
PtoUGT74E2-Probe	CTTTGTTGTTTGGCCCCATGCAAAT	Probe for WTTSSCSS
PtoUGT74E2-Pro-F	GTCGACGTGATTGCTGATGAGT	Promoter cloining for PtoUGT74E2
PtoUGT74E2-Pro-R	GGATCCCTCTATGTACATCTGAT	Promoter cloining for PtoUGT74E2
PtoUGT74E2-F	ATGGAGAGGGAACAAAAAACCAG	CDS for PtoUGT74E2
PtoUGT74E2-R	AGGTCTCTGATCAGGACTCTTG	CDS for PtoUGT74E2
