# Supplementary figures and images for: Allelic variations of WAK106‐E2Fa‐DPb1‐UGT74E2 module regulate fibre properties in Populus tomentosa
Source: Plant Biotechnol J. 2023 Nov 21;22(4):970–86. doi: 10.1111/pbi.14239 (PMC10955495; doi:10.1111/pbi.14239)

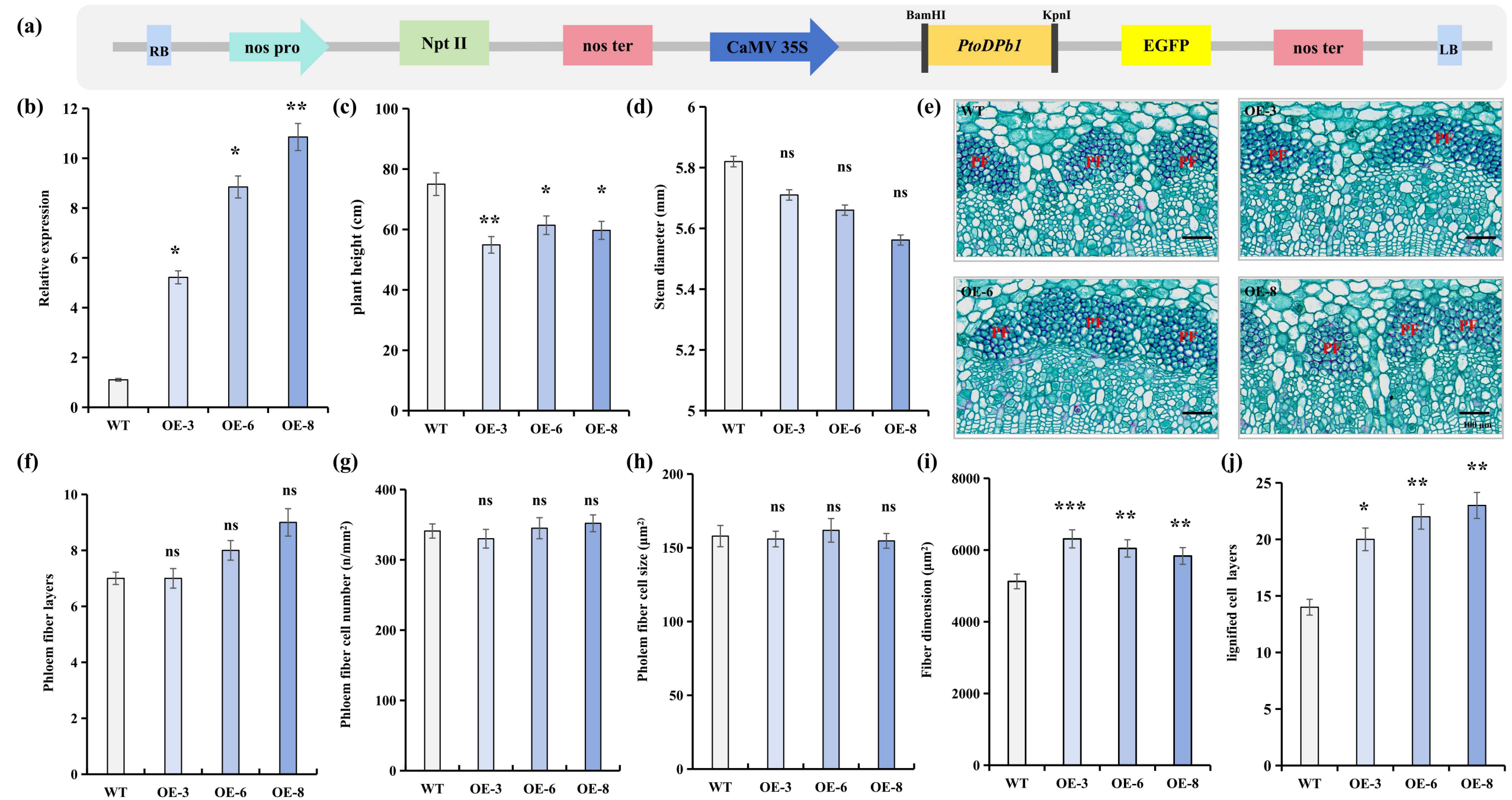

Supplement: Supplementary file 2 — Figure S1 Tissue‐specific expression analysis of PtoDPb1. Figure S2 Sequence alignment of PtoDPb1Hap1 and PtoDPb1Hap2 proteins. Figure S3 The phenotypes of PtoDPb1‐overexpressing lines. Figure S4 Correlation analysis of expression levels of 10 randomly selected differentially expressed genes by the reverse transcription real‐time quantitative PCR (RT‐qPCR) and RNA‐sequencing (RNA‐seq). Figure S5 Estimates of the genetic effects of allelic SNPs in PtoDPb1 on the expression of PtoUGT74E2 and HC traits. Figure S6 Phylogenetic tree analysis of E2F members and DP members. Figure S7 Tissue‐specific expression analysis of PtoE2Fb1, PtoE2Fb2, and PtoE2Fc. Figure S8 The phenotypes of PtoUGT74E2‐overexpressing lines. Figure S9 Significantly associated significant single nucleotide polymorphisms (SNPs) of upstream regulators identified using expression quantitative trait nucleotide (eQTN) mapping. Figure S10 Impact of protein interaction between the PtoE2Fa and PtoWAK106‐PtoDPb1. Table S1 Transcription profiling of RNA‐seq datasets used in co‐expression analysis. Table S2 Details of significant single nucleotide polymorphisms (SNPs) associated with wood property traits and carbohydrate metabolite traits in the association population of Populus tomentosa. Table S3 The 68 connected genes with PtoDPb1 using weighted gene co‐expression network analysis (WGCNA). Table S4 The 36 overlapping genes detected using weighted gene co‐expression network analysis (WGCNA) and RNA‐sequencing (RNA‐seq) analysis. Table S5 Downstream genes identified using expression quantitative trait nucleotide (eQTN) mapping. Table S6 Mendelian randomization (MR) results of the relationship of allelic SNPs of PtoDPb1, expression of PtoUGT74E2, and HC traits. Table S7 Upstream regulators identified using expression quantitative trait nucleotide (eQTN) mapping. Table S8 The oligonucleotide sequences of primers used in this study. Method S1 Association population and phenotypic data. Method S2 Weighted gene co‐e [file PBI-22-970-s002.zip › pbi14239-sup-0003-Corrected Supplementary Figure 3.pdf]

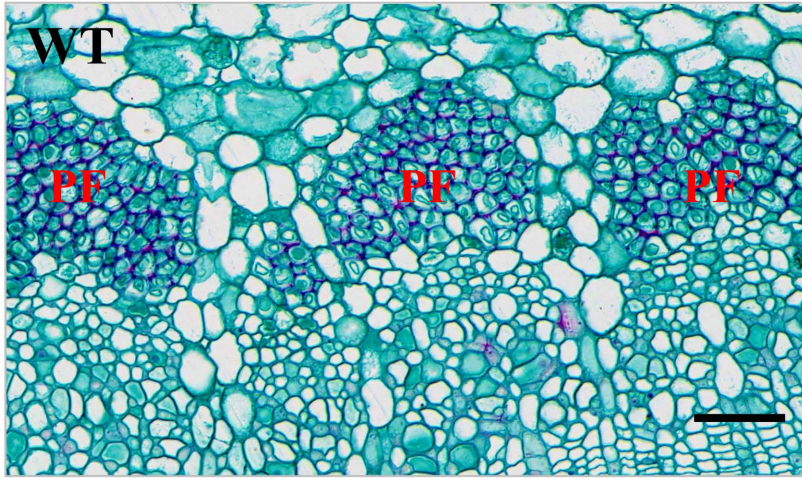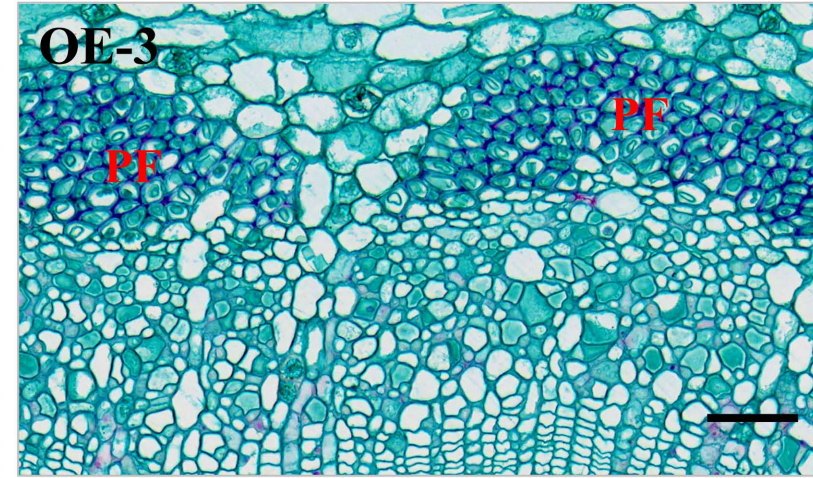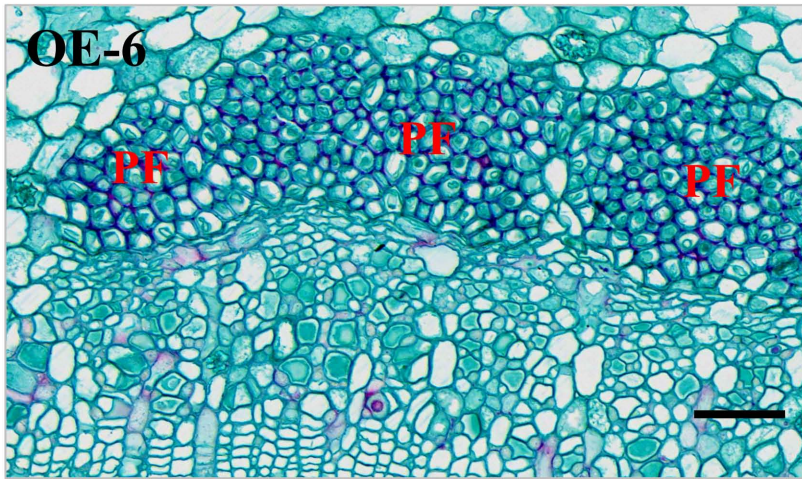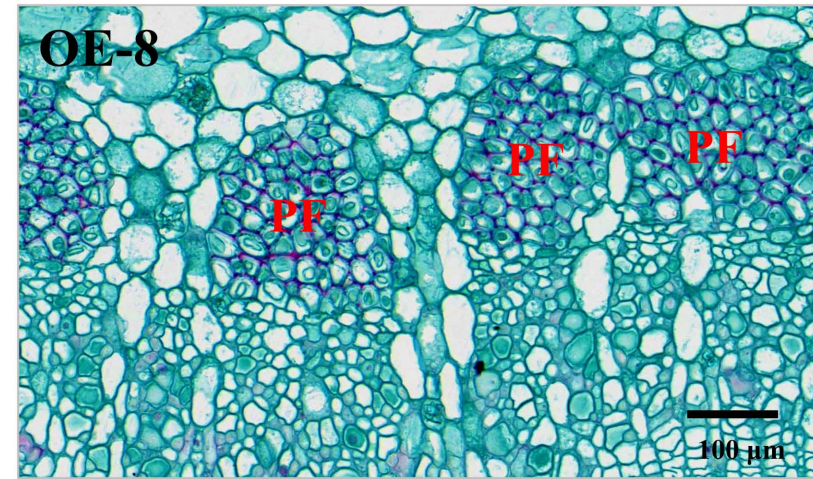

Supplement: Supplementary file 2 — Figure S1 Tissue‐specific expression analysis of PtoDPb1. Figure S2 Sequence alignment of PtoDPb1Hap1 and PtoDPb1Hap2 proteins. Figure S3 The phenotypes of PtoDPb1‐overexpressing lines. Figure S4 Correlation analysis of expression levels of 10 randomly selected differentially expressed genes by the reverse transcription real‐time quantitative PCR (RT‐qPCR) and RNA‐sequencing (RNA‐seq). Figure S5 Estimates of the genetic effects of allelic SNPs in PtoDPb1 on the expression of PtoUGT74E2 and HC traits. Figure S6 Phylogenetic tree analysis of E2F members and DP members. Figure S7 Tissue‐specific expression analysis of PtoE2Fb1, PtoE2Fb2, and PtoE2Fc. Figure S8 The phenotypes of PtoUGT74E2‐overexpressing lines. Figure S9 Significantly associated significant single nucleotide polymorphisms (SNPs) of upstream regulators identified using expression quantitative trait nucleotide (eQTN) mapping. Figure S10 Impact of protein interaction between the PtoE2Fa and PtoWAK106‐PtoDPb1. Table S1 Transcription profiling of RNA‐seq datasets used in co‐expression analysis. Table S2 Details of significant single nucleotide polymorphisms (SNPs) associated with wood property traits and carbohydrate metabolite traits in the association population of Populus tomentosa. Table S3 The 68 connected genes with PtoDPb1 using weighted gene co‐expression network analysis (WGCNA). Table S4 The 36 overlapping genes detected using weighted gene co‐expression network analysis (WGCNA) and RNA‐sequencing (RNA‐seq) analysis. Table S5 Downstream genes identified using expression quantitative trait nucleotide (eQTN) mapping. Table S6 Mendelian randomization (MR) results of the relationship of allelic SNPs of PtoDPb1, expression of PtoUGT74E2, and HC traits. Table S7 Upstream regulators identified using expression quantitative trait nucleotide (eQTN) mapping. Table S8 The oligonucleotide sequences of primers used in this study. Method S1 Association population and phenotypic data. Method S2 Weighted gene co‐e [file PBI-22-970-s002.zip › pbi14239-sup-0004-Corrected Supplementary Figure 3e.pdf]
